# Contribution of transcriptional regulation to natural variations in *Arabidopsis*

**DOI:** 10.1186/gb-2005-6-4-r32

**Published:** 2005-03-15

**Authors:** Wenqiong J Chen, Sherman H Chang, Matthew E Hudson, Wai-King Kwan, Jingqiu Li, Bram Estes, Daniel Knoll, Liang Shi, Tong Zhu

**Affiliations:** 1 Torrey Mesa Research Institute, Syngenta Research and Technology, 3115 Merryfield Row, San Diego, CA 92121, USA; 2 Diversa Corporation, 4955 Directors Place, San Diego, CA 92121, USA; 3 Department of Crop Sciences, University of Illinois, 1101 W. Peabody, Urbana, IL 61801, USA; 4 Syngenta Biotechnology, 3054 Cornwallis Road, Research Triangle Park, NC 27709, USA; 5 Institut für Allgemeine Botanik, Universität Hamburg, Ohnhorststrasse 18, 22609 Hamburg, Germany

## Abstract

**Background:**

Genetic control of gene transcription is a key component in genome evolution. To understand the transcriptional basis of natural variation, we have studied genome-wide variations in transcription and characterized the genetic variations in regulatory elements among *Arabidopsis *accessions.

**Results:**

Among five accessions (Col-0, C24, L*er*, WS-2, and NO-0) 7,508 probe sets with no detectable genomic sequence variations were identified on the basis of the comparative genomic hybridization to the *Arabidopsis *GeneChip microarray, and used for accession-specific transcriptome analysis. Two-way ANOVA analysis has identified 60 genes whose mRNA levels differed in different accession backgrounds in an organ-dependent manner. Most of these genes were involved in stress responses and late stages of plant development, such as seed development. Correlation analysis of expression patterns of these 7,508 genes between pairs of accessions identified a group of 65 highly plastic genes with distinct expression patterns in each accession.

**Conclusion:**

Genes that show substantial genetic variation in mRNA level are those with functions in signal transduction, transcription and stress response, suggesting the existence of variations in the regulatory mechanisms for these genes among different accessions. This is in contrast to those genes with significant polymorphisms in the coding regions identified by genomic hybridization, which include genes encoding transposon-related proteins, kinases and disease-resistance proteins. While relatively fewer sequence variations were detected on average in the coding regions of these genes, a number of differences were identified from the upstream regions, several of which alter potential *cis*-regulatory elements. Our results suggest that nucleotide polymorphisms in regulatory elements of genes encoding controlling factors could be primary targets of natural selection and a driving force behind the evolution of *Arabidopsis *accessions.

## Abstract 2

Among five accessions 7,508 probe sets with no detectable genomic sequence variations were identified on the basis of the comparative genomic hybridization to the *Arabidopsis* GeneChip microarray, and used for accession-specific transcriptome analysis, identifying 60 genes that were differentially expressed in different accession backgrounds in an organ-dependent manner. Correlation analysis of expression patterns of these 7,508 genes between pairs of accessions identified a group of 65 highly plastic genes with distinct expression patterns in each accession.

## Background

Transcription of mRNA from DNA and subsequent translation of mRNA into protein transform genetic blueprints into cellular functions. This process of gene expression and regulation plays a key role in determining the fitness of the genome, through the production of different proteins in different cells and at different times. Therefore, in addition to genome composition and structure, regulation of gene expression is also a key component in development and evolution [1].

The importance of regulatory genes during evolution is well recognized [2]. For example, major differences in axial morphology consistently correlate with a difference in spatial regulation of *Hox *gene expression [3,4]. In addition, a *cis*-regulatory element has functionally diverged during the course of bird and mammal evolution and has resulted in different gene-expression patterns between these two taxa [3,4]. Recently, many studies have suggested that *cis*-regulatory regions of regulatory genes and their downstream target genes might be a major driving force behind evolutionary changes in humans [5]. In plants, evidence for the importance of variations in upstream regulatory regions in the evolution of plant form have also been described. Polymorphisms in an upstream regulatory region of the *teosinte branched1 *gene have been implicated in the domestication of maize [6], and changes in the promoter region of *ORFX *may associate with increases in fruit size during tomato domestication [7,8].

Despite its potential importance, the genetic basis of *cis*-regulatory evolution is poorly understood. Stone and Wray [1] suggested the following reasons: first, the lack of information on sequence variations in the regulatory regions, and lack of association between the degree of coding sequence divergence and the change in gene expression [9]; second, the lack of experimental data from gene-expression analyses to support sequence variation analyses; and third, the lack of a conceptual framework for understanding regulatory evolution that could guide empirical studies. Therefore, to better understand *cis*-regulatory evolution and its implications for genome stability and dynamics, an essential step is to identify sequence variations in the regulatory regions of regulatory genes and downstream target genes on a genome-wide scale, and establish the correlations between gene-expression variations and regulatory sequence divergence. However, few studies have attempted to correlate molecular studies of the evolution of *cis*-regulatory genotype with that of phenotype [10].

Naturally occurring phenotypic differences such as leaf shape or biomass among different *Arabidopsis *accessions [11] have recently become used as resources to study gene function, which traditionally has been studied through mutagenesis and phenotypic characterization of genetic variants [12]. Differences in transcriptional regulation have the potential to contribute substantially to such phenotypic differences among accessions. Thus, it is important to understand the extent to which evolutionary differences between accessions are the result of regulatory polymorphisms causing alterations in transcription, as opposed to coding-region polymorphisms that alter the function of gene products. Although transcriptional profiling has been applied to study the transcriptome differences within or among species using both Affymetrix oligonucleotide GeneChip microarrays and cDNA microarrays [13-15], a recent study from Hsieh *et al*. [16] showed a strong species-by-probe interaction effect when using Affymetrix GeneChip microarray for such inter-species transcriptome analysis. Species differences in hybridization signal strength from a probe set can reflect both sequence differences between probes and their hybridizing targets, and differences in abundance of the mRNA. Therefore, comparative transcriptome analysis of different species or accessions is difficult to interpret without controlling for the effect of coding DNA polymorphism before assaying for differences in transcript abundance.

The objectives of this study are to develop a reliable method for comparing transcriptomes among samples with different genetic backgrounds, to identify differences in transcriptomes among different genetic lines, and to understand the regulatory mechanisms responsible for gene-expression differences by analyzing their predicted promoters. To accomplish these goals, we have adopted a new analysis strategy to analyze the transcriptome variations in five *Arabidopsis *accessions. Our results suggest that genes with functions involved in signal transduction, transcription and stress response are the primary targets for natural selection. This study should shed light on the field of plant evolutionary genomics by furthering our understanding of how the two-way evolutionary interactions between genomic polymorphisms and transcriptional regulatory mechanisms contribute to shaping the evolution of genome.

## Results

### Strategy for comparing gene expression among accessions

The GeneChip microarray used in this study contains approximately 8,700 probe sets for 8,300 *Arabidopsis *genes, which covers about one-third of the genome of accession Col-0 (ecotype Columbia) [17]. Both perfect match (PM) and mismatch probes of the majority of the probe sets on this GeneChip microarray are able to cross-hybridize to genomic targets from other accessions; however, the hybridization signals are affected by any sequence polymorphisms between the probes and the targets [18]. With the standard Affymetrix algorithms (MAS4.0 or MAS5.0) polymorphisms between the hybridizing mRNA samples are likely to invalidate the assumptions underlying the perfect-match mismatch signal subtraction step, leading to inaccurate measurements of the transcript levels, and thus preventing accurate comparisons of the transcriptomes among different accessions.

**Figure 1:**
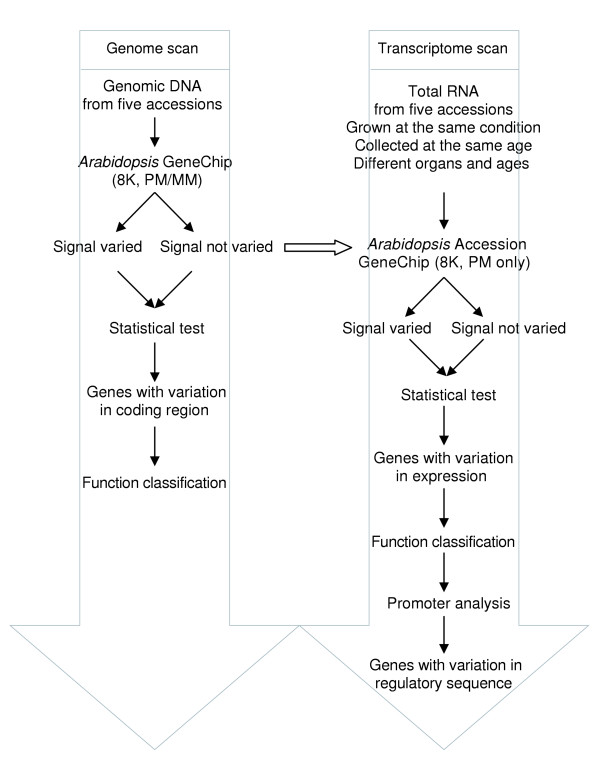
Schematic diagram of the data analysis process. A genome scan (left panel) was used to identify probe sets corresponding to the genes that were highly polymorphic or less polymorphic in gene coding regions among the five accessions. Genes with polymorphic sequences were functionally categorized. Probe sets corresponding to the less polymorphic genes were used for a transcriptome scan of various accessions (right panel). Genes transcribed at different levels in different accessions were identified and analyzed.

To address these issues, we selected for the comparative transcriptome analysis PM probes that hybridize similarly to the genomic targets of test accessions (Figure 1). Briefly, genomic DNAs from different accessions were fragmented, labeled and hybridized to the *Arabidopsis *GeneChip microarrays [19]. The hybridization signals from the PM probes were summarized into genomic DNA hybridization indices (gDHI) using the PM-only model [20] to avoid the complication of the array mismatch probes. The coefficient of variance (CV) of the gDHI among the five accessions used in this study for each probe set was used to determine whether there was sufficient genomic sequence difference among the different accessions to substantially alter hybridization to the oligonucleotide probes. Probe sets were ranked on the basis of their CV and those with the largest CV (CV ≥ 0.20) were eliminated (see Additional data files 1  and 8). The cutoff value was chosen on the basis of the overall mean and standard deviation of the CV from genomic DNA hybridization (mean + standard deviation). For the further comparative transcriptome analysis, 7,736 probe sets with CV less than 0.20 were selected.

To measure the consistency of our probe set selection in this procedure, the reproducibility of the comparative genomic hybridization experiments was determined by labeling and hybridizing the same genomic DNA onto two different microarrays in parallel. The results were highly reproducible and only a small fraction of genes showed twofold or greater difference in hybridization signals between the two replicated experiments: 0.1% between the Col-0 replicates, 0.02% between the L*er *replicates, 0.2% between the C24 replicates, 0.01% between the NO-0 replicates, and 0% between the WS-2 replicates. These results are consistent with the average reproducibility for other genomic DNA labeling and hybridization experiments in *Arabidopsis*, and similar to the results from reproducibility studies for RNA detection using the same GeneChip microarray [17].

### Comparative analysis of transcriptome of different accessions and its validation

Transcription profiles of different organs at different developmental stages (see Additional data file 2) were compared among the five accessions using the following strategy. First, the PM-only model was used to estimate the raw RNA hybridization index (rRHI), to reduce the complication of the array mismatch probes. Second, gDHIs were used to normalize rRHI to remove contributions from sequence variations due to undetected single feature polymorphisms (SFPs) in probe sets. The normalized RNA hybridization index (nRHI), calculated by dividing the rRHI of each probe set by the corresponding gDHI of a particular accession, is used to represent the relative transcript level of the target gene. Third, all the genes were ranked on the basis of their nRHI values, and the lowest 5% were chosen as the cutoff value for background. Genes with an nRHI value less than the cutoff value across all the RNA samples from at least one accession were eliminated from further analysis. By this method, genes whose transcripts could not be detected or were close to the background level were excluded. Fourth, the nRHI values of the 7,508 genes after step 3 were used for statistical analyses, for calculating the Pearson correlation coefficient between all possible pairs of accessions (10 pairs from pairwise comparison of five different accessions) for each gene, and for cluster analysis [21].

To validate variations in transcript abundance detected by the GeneChip microarray through heterologous hybridization using our strategy, quantitative reverse transcription PCR (RT-PCR) using accession-specific primers and probes was performed. Table 1 compares nRHI of 13903_at (At3g54050) and 17392_s_at (At3g53260), measured by the GeneChip microarray and the quantitative RT-PCR in 18 different samples. In general, the quantitative RT-PCR results agreed with the GeneChip microarray results, and confirmed the expression differences of these two genes between accessions Col-0 and C-24. The correlation coefficient between the results of the GeneChip microarray and quantitative RT-PCR is 0.93 for 13903_at, and 0.82 for 17392_s at. As expected, those probe sets with probes cross-hybridizing with genes in a family, such as 17392_s_at, correlated less strongly with accession-specific quantitative RT-PCR.

In addition, nRHI of 12 randomly selected genes with various expression patterns was also validated by quantitative RT-PCR. Some of them did not show different expression levels, and others did show a difference between the flowers of Col-0 and those of L*er*. As shown in Table 2, the results from the quantitative RT-PCR analysis were generally consistent with the nRHI regarding the trend of the change for each gene between Col-0 flower and L*er *flower. There are two exceptions (16892_at and 20545_at), which showed slightly reduced expression in L*er *flower as compared to Col-0 from the GeneChip microarray experiments, but showed an opposite trend of expression from Taqman data. In addition there are a few examples (14172_at and 17860_at), which showed a less than twofold difference from the GeneChip microarray experiments, but slightly higher than twofold differences (14172_at: 2.05-fold, 17860_at: 2.26-fold) from RT-PCR. The slight inconsistency between the GeneChip microarray results and the RT-PCR results may result from the difference in detection technology, and associated sensitivities, between the two methods. It also indicates that definition of significance using twofold change is not appropriate for this experiment. Nevertheless, the results from this extensive validation study using accession-specific primers and probes support our analysis strategy used for transcription analysis of different accessions in both sensitivity and specificity aspects.

To assess the residual interference from sequence variations between targets and probes within the probe sets used for comparative transcriptome analysis, for each sample, we compared the overall transcriptome profiles by calculating Pearson correlation coefficient between rRHI and nRHI for selected probe sets and all probe sets including those probe sets detecting significant difference in genomic hybridization. A general consistency for each sample was observed (see Additional data files 3 and 9). However, the inclusion of the probe sets detecting difference in genomic hybridization reduces the Pearson correlation coefficients between rRHI and nRHI (see Additional data file 3), demonstrating a greater degree of interference from sequence variation in those probe sets. Data from Tables 1 and 2 also showed examples of high correlation between the rRHI and nRHI. When these data were compared to the data from accession-specific quantitative RT-PCR, the correlation coefficients were slightly different: 0.92 (rRHI) and 0.93 (nRHI) for 13903_at, and 0.80 (rRHI) and 0.82 (nRHI) for 17392_at. These results indicate that the probe sets selected for the comparative transcriptome analysis have a low level of interference, and can be utilized to measure the transcript abundance in the five accessions.

### General similarities of transcriptional profiles among accessions from various organs at different stages

**Figure 2:**
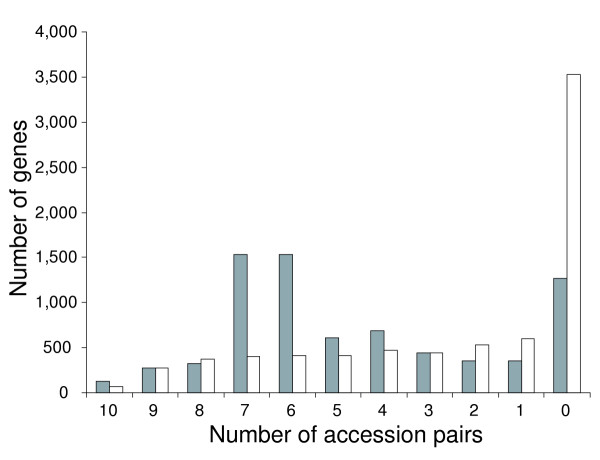
Correlation analysis of expression patterns of genes among the five accessions. A histogram based on the number of genes in each of the 11 groups in Table 3 that have Pearson correlation coefficients less than 0.5 in a given number of pairwise comparisons (see Table 3 for explanation). The white bars indicate the numbers of genes from the experimental datasets, and the gray bars indicate the average numbers of genes from the 10 permuted datasets, as described in Materials and methods.

As shown in Table 3 and Figure 2, among the 7,508 genes whose expression was above the cutoff value in at least one of the RNA samples, the expression patterns of most of the genes (5,985) were correlated (*r *> 0.5) in at least five pairwise comparisons (gray bars), indicating that the expression patterns for most genes from different accessions share some similarity. To test whether the high correlation in expression patterns among different accessions was likely to be obtained by chance, we randomly permuted the RNA samples from the same organs of five different accessions (see Materials and methods for details). The number of genes whose expression did not correlate at *r *> 0.5 for any pair of accession comparisons increased significantly (Figure 2, white bars) from a total of 65 in the original data to 130 (group 10 in Figure 2), and the number of genes whose expression did correlate for all pairs of accession comparisons decreased significantly, from 3,532 in the original data to 1,266 in the permuted data. Because of the close relationship of the five accessions chosen in this study, these data suggest, as expected, that the tissue-specific gene-expression patterns are more consistent between accessions of a single species than any accession-specific patterns between organs.

**Figure 3:**
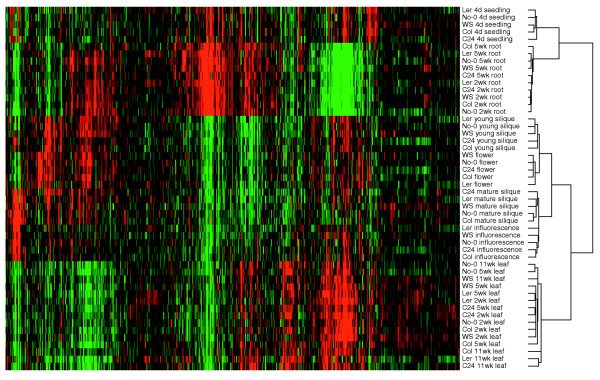
Relationships among the five *Arabidopsis *accessions based on their expression patterns in different organs at various developmental stages. The normalized expression values, obtained by dividing the mRNA expression indices of each organ of one accession by the intensity indices in genomic DNA hybridization for that particular accession, were log_2_-transformed and subjected to cluster analysis. The yellow vertical lines separate the whole cluster into three subclusters, the root cluster, the vegetative leaf cluster, and the reproductive organ cluster.

We used by cluster analysis of the nRHI data to further analyze relationships among the accessions on the basis of the transcriptome profiles (Figure 3). The overall relationships among all samples confirmed that the expression differences among the accessions were small, as the gene-expression differences were greater across different organs of the same accession than that across different accessions in the same organ (Figure 3). Two clusters emerge from the experimental tree: a cluster of axis-origin organs, including roots and young seedlings, and a cluster of auxiliary organs, including vegetative leaves, flowers and siliques (reproductive leaves) and the associated inflorescences (Figure 3). The axis cluster consisted of roots from two different developmental stages - 2 weeks and 5 weeks - as well as 4-day-old seedlings, which are mainly composed of root tissues. The cluster of auxiliary organs could be further divided into two subclusters, one for the vegetative leaves, and one composed of organs originating from the reproductive leaves. Within an organ, especially for leaves, however, variations were contributed by both developmental differences and accession differences. These relationships, as illustrated in Figure 3, were supported by bootstrap analysis [22]. One hundred datasets, each containing the same number of genes, were generated from the original dataset by random sampling with replacement. The bootstrap results confirmed the robustness of the cluster results at the top two levels of the dendrogram (Figure 3).

### Accession-specific gene expression during development

**Figure 4:**
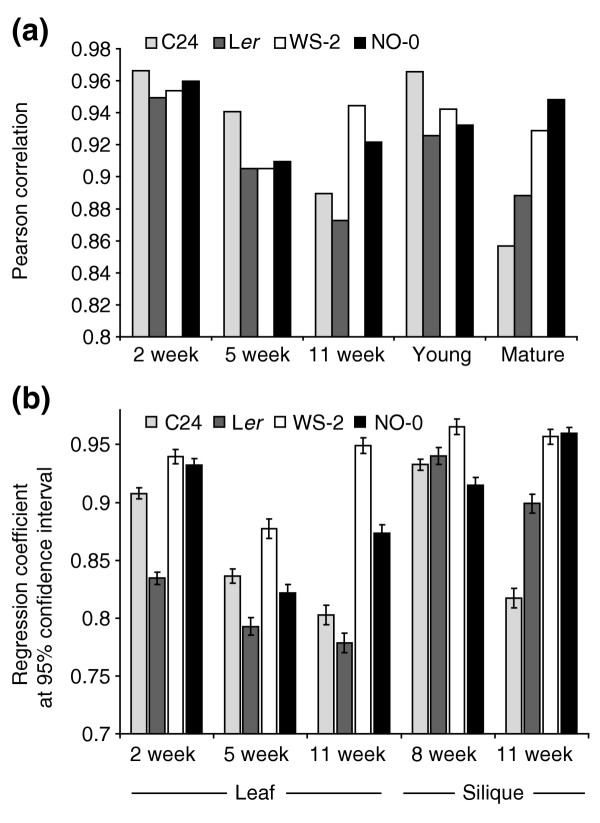
Correlations in transcription among five accessions during leaf and silique development. **(a) **The Pearson correlation coefficient for a given sample was calculated with nRHI for all the genes from each accession and the reference accession Col-0. Each bar represents the correlation of a particular accession as compared to Col-0 in the sample group. Note the common trend in reduction of the correlation during leaf and silique development for each organ. **(b) **The regression coefficient for a given sample was calculated with nRHI for all the genes from each accession (*Y*-values, regressor) and the reference accession Col-0 (*X*-values, predictor). Each bar represents the regression coefficient of a particular accession as compared to Col-0 in the sample group. The regression coefficient (*b*) was calculated as *b *= (Σ*X*_*i*_*Y*_*i *_- (Σ*X*_*i*_)(Σ*Y*_*i*_)/*n*)/(Σ*X*_*i*_^2 ^- (Σ*X*_*i*_)^2^/*n*), where *n *is the total number of genes in either Col-0 or the sample to be compared (7,508 in this case). The error bar indicates the upper or lower limit of the 95% confidence interval for each of the given regression coefficients. The 95% confidence interval was calculated as *b *± *t*α_(2)_, _(*n*-2)_S*b*, where *t*α_(2)_, _(*n*-2) _is the *t *critical value at α = 0.05, two-tail, df = 7,506, and S*b *is the standard deviation of *b*.

Although in general, the gene-expression patterns from the same organs of different accessions were similar, the correlation tends to get worse towards late development (Figure 4). The differences observed among the five accessions in late development could be due to the following reasons: biological noise (individual variation) within each accession during the sampling of biological materials; developmental differences among different accessions; and accession-specific differences due to default regulatory programming. It is unlikely that the differences are due to the sampling noise, as these noises will become undetectable by extensive pooling of biological materials in this study.

The phenotypic differences, especially during late plant development, such as leaf shape, size and flowering time, prompted us to search for genes whose expression is different among different accessions. To identify genes that represent accession-specific difference, and to differentiate them from the genes which could possibly reflect the developmental differences of these five accession plants at the same age grown under the same conditions, we used the one-way analysis of variance (ANOVA) to analyze nRHI data of 2-, 5-, and 11-week-old leaves from the five accessions. Here we treated samples from 2-, 5-, and 11-week-old leaves as three leaf replicates for each accession, thus the only factor we are analyzing is 'accession' which has five levels in this study (see Additional data file 4).

**Figure 5:**
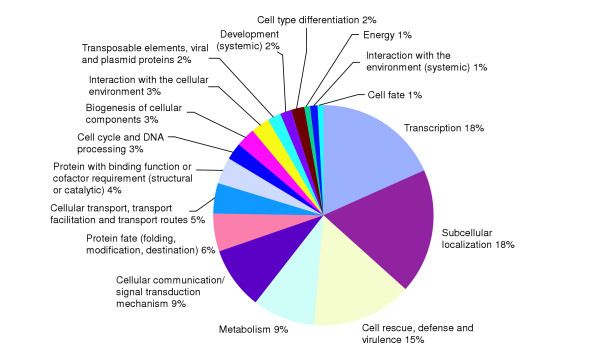
Functional distribution of genes that are differentially regulated in leaves of the five accessions. Fifty-eight genes, identified by one-way ANOVA analysis, were subjected to MIPS functional classification based on their annotations.

On the basis of ANOVA, 1,525 genes were found to have *p*-values less than 0.01 (false discovery rate or FDR = (7,508 × 0.01)/1,525 = 4.9%). Bonferroni correction was further applied for the strong control of family-wise type I error rate (FWER). As shown in Table 4, 58 genes were thus selected, which potentially represent the genes with differential expression among the leaves from the five accessions (*p *< 0.05). These genes were then functionally classified according to the Munich Information Centre for Protein Sequences (MIPS) functional classification. As shown in Figure 5, these 58 genes encode products with diverse functions. Besides those proteins with unknown function, the top five categories contained genes with possible functions in transcription (18% vs 9% for all the genes on the chip), subcellular localization (18% vs 11% overall), stress/defense response (15% vs 6% overall), metabolism (9% vs 18% overall) and signal transduction (9% vs 9% overall). Compared to the overall distribution for all the genes on the chip among different functional categories, genes involved in transcription, subcellular localization and stress/defense response are enriched in this group (*p *≤ 0.008, *p *≤ 0.018, and *p *≤ 0.004, respectively). Eight genes encoding putative transcriptional regulators, including Dof zinc-finger transcription factors, HD-zip transcription factor Athb-8, and MADS-box containing proteins, were included within this group of 58 genes. Genes involved in stress/defense responses include ones that encode disease-resistant proteins such as those of the TIR-NBS-LRR class, enzymes involved in secondary metabolism, and proteins involved in detoxification.

### Organ-specific gene expression in different accessions

In addition to identifying accession-specific genes, we were also interested in determining if there were genes whose expression is regulated by accession-by-organ interaction. In other words, we tried to test if the accession effect on gene expression is organ/development dependent. To address this question, two-way ANOVA analysis was performed. In one case, two samples from 2- and 5-week-old leaves, and two samples from 2- and 5-week-old roots were treated as replicates. In this two-way ANOVA study, the two factors are 'accessions' and 'organs'. For the 'accession' factor, there are five levels. For the 'organ' factors, there are eight levels (see Additional data file 4). The total mean squares for all the genes due to organ difference was 13,182.91 (df = 7), much greater than the total mean squares due to accession difference, which was equal to 2,936.21 (df = 4), consistent with our previous observation from the cluster analysis (Figure 3). The total mean square due to accession-by-organ interaction was only 436.00 (df = 28), suggesting that the effect of accession-by-organ interaction on gene expression might be small. Among the 296 genes that were found to have *p*-values less than 0.01 (FDR = 25.36%), 60 were further selected following Bonferroni correction to control the type-I error rate (Table 5), and subjected to functional classification.

**Figure 6:**
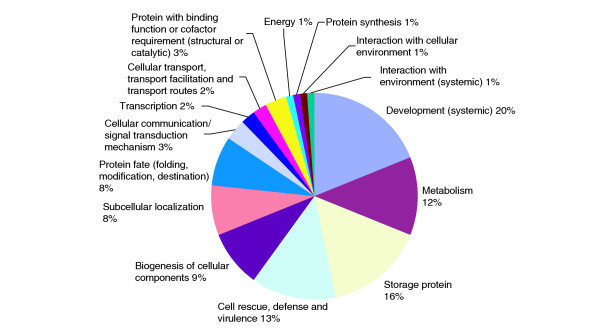
Functional distribution of genes that are differentially regulated by accession-by-organ interactions. Fifty-two genes, identified by two-way ANOVA analysis, were subjected to MIPS functional classification based on their annotations.

As shown in Figure 6, the top five categories contained genes with possible functions in plant development/embryonic development, metabolism, seed storage, stress/defense response and biogenesis of cellular components such as cell walls. Compared to the overall distribution for all the genes on the array among different functional categories, genes involved in plant development/embryonic development and in seed storage are enriched in this group (*p *≤ 0.001 for both categories), suggesting that the differential gene expression in different accession backgrounds might be more profound during late plant development. In contrast to a higher percentage of genes encoding transcription factors, which are differentially expressed in leaves of different accessions, much fewer such genes were found in this group.

### Genes with expression patterns that vary greatly among accessions

**Figure 7:**
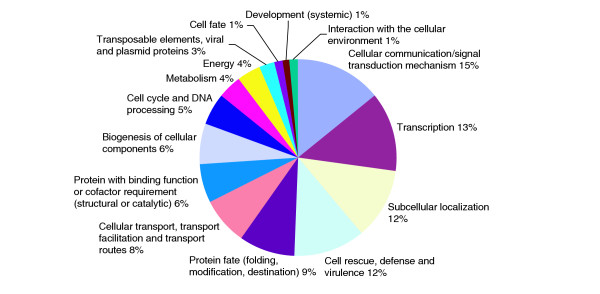
Functional distribution of the 65 most plastic genes. The 65 most plastic genes identified from the expression correlation analysis, whose correlation coefficients are less than 0.5 in all 10 pairwise compared accessions, were subjected to MIPs functional classification based on their annotations.

For each gene, the expression pattern reflects the relative abundance of its mRNA in different RNA samples, which is determined by a combination of environmental and developmental factors. Thus the differences in gene-expression patterns from different accessions reflect the different responses of each accession to these factors. To identify genes whose expression is highly sensitive to various environmental and developmental stimuli, and to further understand the differential regulatory mechanisms among accessions, genes with distinct expression patterns in different accessions were identified by their correlation coefficients between every two accessions in the Pearson correlation coefficient matrix (Figure 2), using 10 data points from the corresponding 10 organs of each accession (see Additional data file 5 for an example). Of these, 65 genes had correlation coefficients less than 0.5 in all 10 pairs of accession comparisons (Table 6), 271 genes had correlation coefficients less than 0.5 for nine pairs of comparisons, and 376 genes had correlation coefficients less than 0.5 for eight pairs of comparisons (Figure 2). As shown in Figure 7, genes belonging to functional categories of signal transduction, transcription, subcellular localization, stress/defense response and protein fate (folding, modification, destination) are among the top five functional categories in this group, whereas the proportion of genes belonging to the transcription functional category is slightly higher (13% for this group and 9% for the overall group). Genes involved in transcription included different types of transcription factor genes, such as *bHLH*, EREBP-like, and several zinc-finger transcription factor genes. Genes whose products are required for other functions related to the control of mRNA level, such as chromatin remodeling or RNA processing (for example, the mRNA capping enzyme and the chromatin-remodeling factor CHD3 (PICKLE)) were also included in this group (Table 6). The stress-responsive genes included those for the putative heat-shock protein DnaJ and the α-jacalin-like lectin, a relative of which has been shown to be salt-stress-inducible in rice [23]. A number of genes, whose products are protein kinases and are likely to be involved in cell signaling pathways, were also included in this 65-gene list.

### Regulatory sequence polymorphisms could account for the gene-expression differences among accessions

To test whether the accession-dependent differences we observed were caused by polymorphisms in regulatory sequence, we sequenced the promoters and coding regions of seven genes selected from genes with Pearson correlation coefficients less than 0.5 in at least five pairwise comparisons among the five accessions discussed here (plus seven additional accessions, RLD-1, Ag-0, Bs-1, Cvi-0, Es-0, Gr-1, Mt-0 and Tsu-0, to obtain a better estimate of relative substitution rates). We identified a total of 167 polymorphic bases in one or more of the five accessions (316 in all 12) across 24.9 kilobases (kb) of promoter and coding sequence. The polymorphism rate among all five accessions in regulatory (promoter) sequence was 8.06 per kilobase, compared to 10.5 per kilobase in introns and 4.08 in exon sequence (Table 7), indicating that regulatory sequence is the repository for substantially more genetic variation than coding sequence. Details of these polymorphisms are described in Additional data file 6.

We then analyzed the promoter sequences of the seven genes selected for further study of sequences matching known plant *cis*-regulatory elements (see Materials and methods) to determine whether any of the polymorphisms altered sequences corresponding to known *cis*-regulatory motifs in the promoters. We found that a total of 44 out of the 61 polymorphisms among the seven genes fully sequenced in the five accessions caused alterations in sequences that matched known *cis*-regulatory motifs (details of all these changes are provided in Additional data file 6). For example, the putative RING-finger protein At4g10160 is one of three genes encoding proteins in this family that we resequenced in the target accessions. In Col-0, the promoter of At4g10160 contains a CAACA element at -164, which is absent in all other accessions as the result of a sequence polymorphism. This element is the binding site for the transcription factor RAV1. RAV1 belongs to the AP2/EREBP transcription factor family, members of which are involved in various aspects of plant development as well as in plant response to environmental stresses [24]. When the expression profiles of this gene were considered, the lowest three correlation coefficients between any of the pairs of accessions were those between Col, Ws, No-0 and L*er *(*r *= -0.045, -0.168 and 0.201 between the pairs Col/C24, L*er*/WS and Ler/No-0, respectively).

Not all of the transcription difference is associated with altered known *cis*-elements. For instance, the gene for the PHYB photoreceptor, At2g18790, was also differentially expressed among accessions. There were several polymorphisms in the promoter sequence, most of which were specific to the Ws accession (a natural mutant in another phytochrome gene, *PHYD *[25]). These polymorphisms included two mutations that both altered *cis*-regulatory elements (AAAGAA to ATAGAA at -965, and GGTTTATT to GCTTTATT at -445) known to be involved in the regulation of another phytochrome gene [26]. These polymorphisms could not fully account for the different expression patterns, however, as the Col-0 expression pattern correlated quite well to that for Ws (*r = *0.78), whereas the L*er*/Ws pair correlated very poorly (*r = *0.207). The correlation between Col-0 and C24 was only *r = *0.341. Because Col-0 and C24 had identical sequence throughout the *PHYB *promoter, the difference in expression patterns must be at least partly explained by other factors, such as polymorphisms in enhancers outside the resequenced region, or polymorphisms in the genes encoding regulatory factors that control *PHYB *mRNA levels.

## Discussion

A number of interspecies or interaccession comparative analyses of transcriptomes using GeneChip microarrays have been attempted recently. Brem *et al*. [27] conducted a study in yeast to understand the genetic architecture of natural variation in gene expression using GeneChip microarrays. By comparing the transcriptomes of two yeast strains, the study linked 570 differentially expressed genes between the two parental yeast strains to one or more genetic markers, and further grouped these genes into two categories, the *cis*-acting modulators and *trans*-acting modulators. More recently, two laboratories independently used the *Arabidopsis *GeneChip microarrays to detect transcriptional changes in metal homeostasis genes of *A. halleri*, a closely related species to *A. thaliana *and a natural metal hyperaccumulator [28,29]. These studies successfully demonstrated the potentials of GeneChip microarrays in the studies of biodiversity among *Arabidopsis *accessions and the closely related species, as supported by extensive validations from real-time RT-PCR, and RNA blot experiments. However, these studies were limited to those genes whose mRNAs were expressed at high levels, as they used stringent selection criteria. In addition, the signal differences contributed by the sequence variations between the two species or lines were largely unaddressed.

To apply GeneChip microarrays developed for a model species to monitor transcription in other related accessions or species, and to enable the comparisons of transcriptomes among closely related accessions or species with genetic variations, we developed a new strategy for analyzing transcriptome profiles from GeneChip experiments by heterologous probe-target hybridization (Figure 1).

To minimize the interference from detectable sequence variations between probes selected from one accession and targets from another accession, we identified and selected those probe sets that hybridize similarly to genomic targets from different accessions, and excluded the ones which showed significant difference in their hybridization signals for further analysis. We analyzed the data at the probe set levels using Li Wong's PM-only model, as this algorithm takes probe effect into consideration by proper modeling and summarization of probe-level data into probe set indices [30]. We did not perform our analysis at the probe level, because, first, there are substantial single feature polymorphisms (SFPs) among *Arabidopsis *accessions, as demonstrated between Col-0 and Ler [18]. If we remove all the probes with SFPs, it will reduce the number of available probes in a probe set, thus compromising the quality of the measurements. Second, comprehensive detection of SFPs is not within the scope of this study. The high correlations observed between the rRHI and nRHI suggest those residual sequence variations between probes and targets from different accessions did not substantially affect the comparisons between mRNA level in the different accessions.

Only 986 probe sets (out of 8,722 probe sets) showed substantial difference in genomic DNA hybridization signals from the genomes of the five accessions we investigated (see Additional data file 1). These probe sets, representing the genes with high polymorphism rates, were functionally categorized, and were consistent with the results obtained by the previous study where a number of *Arabidopsis *SFPs were identified by large-scale comparative genome analysis [18]. For example, among the 127 transposon related genes presented on the array, 88 of them were detected as polymorphic among the five accessions. The molecular mechanism that underlies this observation was not clear, although reduced selection pressure for sequence conservation between transposable elements, combined with the mutations that can result from transposition events, may lead to a higher polymorphism rate. Transposable elements are likely to play an important role in shaping the plant genome [31]. In addition to transposon-related genes, genes encoding disease-resistance proteins and kinases were also found to contain SFPs among different accessions.

The specificity of the GeneChip microarray detection was validated experimentally by other methods such as real-time quantitative RT-PCR, using accession-specific primers and probes. Genes for the RT-PCR experiments were selected so that various transcript levels, and various expression patterns during development, were represented, based on the microarray analysis results. The general agreement between the results from GeneChip and the quantitative RT-PCR measurements demonstrate the specificity of the detection in different accessions.

Overall, the transcriptome profiles are relatively consistent during development among the *Arabidopsis *accessions studied. This is supported by the high degree of Pearson correlation coefficients for each expressed gene from every possible pair of compared accessions. It was also supported by cluster analysis of samples from different organs among the five accessions. Seventy-nine percent of the analyzed genes have correlation coefficients greater than 0.5 in at least five pairs of accessions (Figure 2).

Interestingly, similarity in gene expression is not consistent with the similarities in the coding sequence among different accessions. Among the pairwise accession comparisons, we found that the C24/L*er *pair contained the fewest genes whose expressions did not correlate (data not shown). However, this finding was not consistent with the cluster results based on the coding sequence variations, in which the closest accession to C24 was Col (data not shown). This suggests that transcriptional regulation has a significant role in determining natural variations in gene expression, and there might be more difference in gene-regulation mechanisms between C24 and Col-0 than is suggested by the relative similarity of their genomic sequence.

The divergence in transcriptomes and their regulatory mechanism in different accessions become evident from the results of the ANOVA analysis of transcriptomes of 2-, 5- and 11-week-old leaves from the five accessions. It was found that 58 genes showed a statistical difference (*p *< 0.05 after Bonferroni correction) in expression among different accessions, and a higher percentage of these differentially expressed genes encode products in transcriptional regulation, and stress responsive proteins (Figure 5, Table 4). The differences in gene expression in leaves of the five accessions are mainly due to the accession differences, because for those genes the differences at different developmental stages of leaves in each accession are not statistically significant compared with the differences among the five accessions. Although we could not correlate the gene-expression difference with any previous reports on these particular accessions, our data suggest that the differential expression of these genes could reflect adaptive responses to the environmental conditions used in this study. It will be interesting to map these genes to their genetic locations to test if any have been previously linked to quantitative trait loci, thus affecting the phenotypes among different accessions.

The accession differences in transcriptome programming become more obvious towards late development in an organ-specific manner. Sixty genes whose expression might be affected by accession-by-organ interaction during late development were identified. The top five functional categories contained about 71% of genes whose products might be involved in nutrient storage, stress response and plant, especially reproductive, development (Figure 6). As shown in Additional data file 7, the expression of the majority of these genes differed in senescent leaves and mature siliques, suggesting that the transcriptome programs in these organs are more sensitive to different accession backgrounds at late stages, leading to the differential expression of genes involved in late plant development. We could not, however, rule out the possibility that some of these genes might represent the differences in developmental stages for the five accessions around the sample collection time.

To further elucidate regulatory mechanisms that are important for the differential gene expression among different accessions, we have identified 65 genes that showed different expression patterns in the five accessions during development by analyzing the Pearson correlation coefficients from the 10 pairs of compared accessions (Figure 2). The 65 most plastic genes are predominantly those that function in transcription and in stress and defense responses (Figure 7). It has been shown that the expression of many transcription factor genes is sensitive to changes in environmental conditions [32,33]. By examining the expression patterns of these most plastic genes under various environmental conditions [30], such as biotic or abiotic treatments, we found that the expression of a majority of the genes was induced or repressed by various environmental factors, demonstrating their high responsiveness to environmental conditions. These findings suggest that regulatory genes are major targets of natural selection [34], because changes in both the protein structure encoded and gene expression of a limited number of transcription factor genes would result in dramatic phenotypic variations via changes in expression of a large number of downstream genes.

The differences in expression of these genes could arise from multiple mechanisms, such as changes in expression or activity of *trans*-acting regulators, changes in the *cis*-regulatory regions of the corresponding genes, or even epigenetic modification. Previous studies have shown that both regulatory genes and gene promoter regions are subject to selective forces [34] and that promoters are the primary targets of adaptive evolution relative to coding regions [35]. Here we present one such example, At4g10160, which encodes a RING-finger protein. The change in one of the predicted *cis*-elements in the promoter of this gene was consistent with the changes in gene expression. This finding is of particular interest as RING-finger proteins are known to be capable of regulating gene expression and altering developmental patterns and cell proliferation [36,37]. Although this finding requires more experimental validation, it represents a clear example of differential gene-expression mechanisms among different accessions. It is recognized, however, that not all the differences in accession-dependent transcription can be explained by regulatory polymorphisms. The difference in *PHYB *expression between C24 and Col-0 illustrates the complexity of the regulatory mechanism involved in the adaptation of the transcriptome programs. Changes in expression of this gene might be influenced by other factors, such as alterations in the regulatory sequences of genes encoding controlling factors, for example the RING-finger proteins discussed above.

## Conclusion

Using a GeneChip microarray and a strategy validated experimentally by accession-specific quantitative PCR, we compared the transcriptomes of five *Arabidopsis *accessions under identical growth conditions. The detected variations in gene expression among different *Arabidopsis *accessions may be caused by a combination of variations in *trans*-acting factors, or in promoter regions of the variable genes themselves. Using the approach of comparative transcriptome profiling of different accessions, combined with genome sequence information, it is possible to identify polymorphisms putatively associated with the accession-dependent gene-expression patterns, and to link these polymorphisms to the differential expression of genes encoding components of regulatory mechanisms. Mutations of such global consequence are highly likely to have been subject to intense selective pressure during evolution. This could further help in understanding genome and transcriptome dynamics during evolution [38], suggesting that natural selection must not simply act through constantly evaluating the fitness of existing DNA within the genome on a gene-by-gene basis, but also by strongly favoring advantageous polymorphic gene-regulatory mechanisms which arise as a result of rare, but highly significant, genomic mutations that alter the expression patterns of large clusters of genes. Moreover, because phenotypic variation among different accessions probably reflects genetic variation that is important for the plant's adaptation to specific environmental conditions, transcriptome analysis, as a powerful tool for molecular phenotyping, should provide a complementary approach to quantitative trait locus (QTL) analysis for studying the interaction between genetic variation and environment. A potential application of this approach to crop breeding is to identify key regulatory mutations conferring desirable, yet highly pleiotropic, traits in commercial cultivars. Regulatory polymorphisms responsible for these variations may then be readily transferred between cultivars as monogenic traits.

## Materials and methods

### Plant materials, growth conditions and sample processing

Seeds from the five *Arabidopsis *accessions Col-0 (Columbia), C24, WS-2, NO-0, and L*er *(Landsberg *erecta*) were obtained from the *Arabidopsis *stock center (ABRC, Columbus, Ohio). Seeds were geminated in Metro-Mix soil (Scotts-Sierra Horticultural Products) in flats and were grown in controlled-environment chambers CMP4030 (Conviron, Winnipeg, Canada) at 22°C under a 12-hr/12-hr light/dark regime and 80% humidity. Plants received approximately 350 μmol s^-1 ^m^-2 ^of light from two light banks emitting 15.069 lux or 45.2 W m^-2^. Ten different RNA samples from 10 different organ samples, including roots, leaves, flowers and siliques, were collected at different plant ages from each accession (Additional data file 2). All samples were collected from at least 10 individual plants between 11 am and 1 pm and were pooled. RNA was extracted from various organs, which were collected. Genomic DNA was extracted from the 4-week-old leaves. DNase I digestion was used to obtain genomic DNA fragments with average sizes ranging from 25 to 150 nucleotides. DNA fragments were end-labeled using terminal transferase according to Winzeler *et al*. [19]. The *Arabidopsis *Genome GeneChip array (Affymetrix) was used for this study. Details of array features and performance were described previously [15]. The RNA extraction and GeneChip microarray experiments were exactly performed as described by Zhu *et al*. [39].

### Dataset collection, data processing and data analyses

The microarray experiments on genomic DNA hybridization were conducted in replicates for all accessions for the reproducibility analysis. Replicate data from Col-0 and L*er *were used for selecting outliers (see below). All statistical analyses were performed using the BioConductor packages [40] in R [41] and S-plus 6.1 (Insightful). The '.CEL' files were read directly into R and genomic hybridization intensity indices were computed from the individual probes (16-20 for each gene) using the Li-Wong PM-only model [20], which was implemented in the BioConductor package. The outlier genes from either the Col-0 replicates or the L*er *replicates (false positives) were eliminated. The outliers were defined as those genes whose hybridization intensity indices were at least twofold different between the two replicates. For the rest of the genes, the two Col-0 replicates and the two L*er *replicates were averaged separately to obtain a single value, which represents the signal intensities for Col-0 and L*er *genomic DNA hybridization. Then the coefficient of variance (CV) was calculated for each gene on the basis of its genomic hybridization intensity indices from the five accessions. Genes with the highest 11% CV (CV ≥ 0.20) were eliminated from further expression analysis (see Additional data file 1). CV = 0.20 was chosen as the cutoff value on the basis of the following two criteria: it is equal to mean (CV) + 1 standard deviation from genomic DNA hybridization; we tried to exclude as much as possible the genes that could possibly have sequence differences among the five accessions, to ensure less interference when analyzing mRNA expression for the remaining genes. This resulted in 7,736 genes.

Genes for the correlation analysis were selected from the 7,736-gene list from genomic DNA hybridization data. The mRNA expression index for each gene was also computed using the Li-Wong PM-only model [20]. The expression values of the selected genes were normalized by dividing the hybridization indices from RNA hybridization from each organ of a particular accession by the indices from genomic hybridization of this particular accession. The relative expression values for all the genes from all the experiments (7,736 × 50 = 386,800 data points) were sorted and the lowest five-percentile value was used as the cutoff value between noise and true signals. Then, genes whose expression value was below the cutoff value across all the RNA samples from at least one accession were further eliminated. This resulted in 7,508 genes. The normalized expression values were log_2_-transformed and used for the correlation analysis. In addition, this dataset of 7,508 genes was used for permutations in which, for a particular organ at a particular developmental stage, we randomly permuted among the five RNA samples from the five accessions (10 organs × (5 × 4 × 3 × 2 × 1 permutations for each organ) = 1,200 potential combinations), thus preserving the organ-age categorization. Then, for each gene, 10 pairwise comparisons, represented by 10 Pearson correlation coefficients, were made from the five different accessions. The Pearson correlation coefficient for each pair was calculated by using the normalized gene expression values from 10 organs (10 data points) of one accession versus the 10 data points from the other accession (see Additional data file 5 for an example). The number of genes that had *r *< 0.5 in a given pair of compared accessions was calculated and is shown in Table 3 and Figure 2. With the permuted data, the numbers shown in Table 3 and Figure 2 are the averages of the 10-permuted datasets.

Cluster analysis of mRNA expression data was performed with the same list of 7,508 genes used for the correlation analysis. The normalized expression values were then log_2_-transformed, mean centered for each gene across all the samples, and subjected to the self-organizing maps, followed by average linkage hierarchical clustering of both genes and experiments using Cluster and visualized with TreeView to generate Figure 3.

Analysis of variance (ANOVA) of mRNA expression data was performed with the same list of 7,508 genes used for the correlation analysis with functions in S-PLUS 6.1 (InSightful). The normalized expression values were log_2_-transformed and used for the ANOVA analysis. For one-way ANOVA analysis, the three leaf samples from 2-, 5- and 11-week-old leaves were treated as biological replicates, and the general linear model (GLM) is formulated as: expression = accessions + error. For two-way ANOVA analysis only the two leaf samples from 2- and 5-week-old leaves, and two root samples from 2- and 5-week-old roots were treated as biological replicates, and the GLM is: expression = accessions + organs + accessions × organs + error. We excluded the 11-week-old leaves in two-way ANOVA analysis to take into consideration the effect of age on gene expression. We have estimated the variance for each gene in leaves and roots of different accessions using the local pooled error (LPE) method [42], and found that only a small percentage of genes have different variance in other accessions as compared to one in Col-0. As there is no biological replicate for the rest of the organs, we are assuming that the errors for those organs are at similar levels, as estimated from the two leaf and root samples in the two-way ANOVA analysis. Genes with significant *p*-value (*p *< 0.05) after Bonferroni correction were then selected accordingly.

### Statistical analysis for enrichment of MIPS functional categories

To test whether genes representing certain MIPs functional categories are over-represented in the list of statistically significant genes identified from either one-way, or two-way ANOVA, bootstrapping was performed by generating 1,000 control lists from all the genes on the array, each of which contains the same number of genes as contained in the list from either one-way, or two-way ANOVA analysis. Genes in each of the control lists were classified on the basis of MIPS functional categories. Then, for each functional category, a distribution of number of occurrences for that particular functional category from 1,000 control lists was generated, and this distribution was compared to the observed occurrence to determine the *p*-value.

### Validation of the GeneChip microarray data

The genomic sequence for gene 13903_at (At3g54050) and 17392_s_at (At3g53260) from accession C24 was obtained by PCR with genomic DNA from C24, and the following primers based on this gene's coding sequence from Col-0.

13903_at (At3g54050): 5'-primer: 5'-GATCCAATGTACGGTGAGTTTG-3'; 3'-primer: 5'-TGCAT-ATACCATGTAGTCAG-3'.

17392_s_at (At3g53260): 5'-primer: 5'-CAGTTTCTCAAGTTGCTAAG-3'; 3'-primer: 5'-CATTCC-TTGAGACAATCCAT-3'

The PCR product was then sequenced and these sequences were used for designing gene-specific primers and probes for Taqman assay.

The L*er *sequences of genes 12222_s_at (At2g20990), 14097_at (At2g47770), 20561_at (At2g46930), 14634_s_at (At4g27440), 13483_at (At2g25650), 15290_at (At2g20840), 13111_at (At2g38040), 14072_at (At1g67480), 14172_at (At3g54140), 14947_at (At4g37450), 16892_at (At5g45890), 17860_at (At4g27410), 20545_at (At5g27470) were obtained by BLASTing the full-length cDNA sequences or coding sequences of these genes from Col-0 against the L*er *sequences available from TIGR [43]. Top BLAST hits were chosen and sequences common for both Col-0 and L*er *were used to design gene-specific primers and probes for Taqman assay.

Quantitative RT-PCR (Taqman) assays were performed on an ABI Prism 7700 (Applied Biosystems), as previously described [44], using the following gene-specific primers and probe sets:

13903_at_forward primer: 5'-GGTCCAACTGGGAAGCCTTAC-3' 13903_at_reverse primer: 5'-CCGTACAACAAAGTCCTGTGAAAA-3' 13903_at_target probe: FAM-CCAACCAAACTTCCAATGTACCTTGCCGTAMRA.

17392_s_at_forward primer: 5'-GGCTGTGCTTCCAAAGGAAGT-3' 17392_s_at_reverse primer: 5'-GTTAGGAATCGGCGCAGTTC-3' 17392_s_at_target probe: FAM-CTCCCATAAGCTGCTCTAGCCGCTTAMRA.

12222_s_at_forward primer: 5'-GGCTGTGCTTCCAAAGGAAGT-3' 12222_s_at_reverse primer: 5'-GTTAGGAATCGGCGCAGTTC-3' 12222_s_at_target probe: FAM-CTCCCATAAGCTGCTCTAGCCGCTTAMRA.

14097_at_forward primer: 5'-CAACAAAGGAAAACGCGATCA-3' 14097_at_reverse primer: 5'-CGCTACCGTCAGAGACTTGAGA-3' 14097_at_target probe: FAM-AGAGGGCGATGGCGAAACGTGTAMRA.

20561_at_forward primer: 5'-TGGTACTTTGACAGAACAACAGTGAA-3' 20561_at_reverse primer: 5'-TGAAGATGAGATTGTGACATGTTTTG-3' 20561_at_target probe: FAM-CCATTGACTGTCCTTACCCCTGT-TAMRA.

14634_s_at_forward primer: 5'-CGAATACATTGGCGGGTAATG-3' 14634_s_at_reverse primer: 5'-GCCGGCTAAACCCCTCAA-3' 14634_s_at_target probe: FAM-ACCACCGAAGGCGAATCTCGGTGTAMRA.

15290_at_forward primer: 5'-TCCTGGAGCGTATGTTATGTGGTA-3' 15290_at_reverse primer: 5'-CACCCAAACTTCAGAGCACTATCA-3' 15290_at_target probe: FAM-CGCCCTCTTTATCGTGCCATGAGGTAMRA.

14072_at_forward primer: 5'-TGTATGACCCGGATGCTTCA-3' 14072_at_reverse primer: 5'-ACGCAAGAACCAGAGAGTTTGAT-3' 14072_at_target probe: FAM-CAGGCACACAGTGGAAAACGTCTGA-TAMRA.

13111_at_forward primer: 5'-GAGATCAAGAGCATGGTGGAGTT-3' 13111_at_reverse primer: 5'-GGTGACACCAGGCGTTTTG-3' 13111_at_target probe: FAM-CTGAAAGTGGAAACCGCAAAGGCG-TAMRA.

14172_at_forward primer: 5'-GGGTATAGGTCTTGTGGTCTCCAT-3' 14172_at_reverse primer: 5'-ATCAAGCCTGACAACCTCCAA-3' 14172_at_target probe: FAM-TTTGCCATGATCACTGCAGGAG-TAMRA.

14947_at_forward primer: 5'-TCCTAACAGTTACATTGATCTGCATTG-3' 14947_at_reverse primer: 5'-TGGTCGGAGAAGAGATAGGAGATT-3' 14947_at_target probe: FAM-CGTCGCCGGTGTCGGTG-TAMRA.

16892_at_forward primer: 5'-CCGGTTAATGATGAGCAAGCA-3' 16892_at_reverse primer: 5'-CCTCCTTCAATTCCAACGCTAA-3' 16892_at_target probe: FAM-ATGAAGGCAGTGGCACACCAACC-TAMRA.

17860_at_forward primer: 5'-ACGGTGGTTACGATGCGTTT-3' 17860_at_reverse primer: 5'-CCGATTCACATGCCCACTCT-3' 17860_at_target probe: FAM-AGCGGCGGAAGGTGAGGCG-TAMRA.

20545_at_forward primer: 5'-GAGCTTGTGTCTTGTTCCAACTGT-3' 20545_at_reverse primer: 5'-TGCTCTTTTTCTGACCGTATCTGA-3' 20545_at_target probe: FAM-CAGACTACCAGGCTCGCAGGCTTGA-TAMRA.

A standard curve consisting of serial 1:5 dilutions was prepared with RNA concentrations of 50 ng/μl, 10 ng/μl, 2 ng/μl, 0.4 ng/μl, and 0.08 ng/μl. Relative expression levels were interpolated by comparison with standard curves with a correlation coefficient of 0.99 or greater. Relative expression levels were normalized to the expression level of the *Arabidopsis APX3 *gene [44], which was expressed at a constant level. All reactions were performed in triplicate.

### Promoter and polymorphism analysis

Genomic DNA sequencing was used to analyze the polymorphisms in 12 different *Arabidopsis *accessions. Genomic DNA of the accessions Col-0, C24, L*er*, Ws-0, No-0, RLD-1, Ag-0, Bs-1, Cvi-0, Es-0, Gr-1, Mt-0 and Tsu-0 was obtained from tissue supplied by the stock center and used as the template for PCR amplification and sequencing. The sequencing strategy was as follows: using the AGI genome annotation as a guide, a region from 1 kb before the annotated translation start of each gene to 300 bp after the stop codon was amplified by LA-PCR (Long and accurate PCR) from each of the accessions. The PCR product was used directly for sequencing of both strands. Several primers were used to complete the sequencing of the whole gene and the 5' and 3' regions. Using Sequencher software (GeneCodes) the sequences from each accession were put into contiguous alignment for each gene. Sequence variations between the accessions in the promoter region, open reading frame (ORF), intron, exon and 3' UTR were confirmed and recorded. The promoter region was defined as the available sequence (1 kb or more) before the translational start codon, while the intron-exon boundaries were defined using the AGI (Arabidopsis Gene Index) gene models, which were obtained from The *Arabidopsis *Information Resource (TAIR) [45]. Only those differences confirmed in multiple sequencing were determined as polymorphisms. The polymorphism rate in promoters and exons was calculated as the number of bases substituted in any of the sequenced accession plus the total number of different insertion or deletion (indel) events found in all the accession in that sequence region, divided by the length of the available sequence. Alterations in potential *cis*-regulatory elements caused by polymorphisms were detected in the following automated way. The mutant and wild-type promoter sequences were searched for all known plant *cis*-regulatory elements in the databases PLACE [46] and plantCARE [47] using a custom-written PERL script. The lists of *cis*-regulatory elements were compared to find elements created or destroyed by the polymorphisms. This list was then manually edited to remove unlikely candidates for promoter regulatory sequences, such as potential translation initiation sites that were outside the transcribed region, or putative polyadenylation motifs situated in the promoter region.

## Additional data files

The following additional data are available with the online version of this paper. Additional data file 1 is a table showing probe sets representing genes with highly polymorphic coding sequences. Additional data file 2 is a table showing samples used in this study. Additional data file 3 is a table showing correlations between raw and normalized RNA hybridization indices among all 50 samples. Additional data file 4 is a table showing examples of (a) one-way and (b) two-way ANOVA tables from analysis of variance (ANOVA). Additional data file 5 is a table showing an example of the Pearson correlation coefficients matrix for a particular gene obtained from 10 pair-wise comparisons among the five accessions. Additional data file 6 is a table showing the sequence variation in promoter regions that alters *cis*-elements. Additional data file 7 is a table showing mRNA expression of genes identified from two-way ANOVA. Additional data file 8 is a figure showing a histogram of coefficient of variance (CV) based on genomic hybridization intensity indices from the five accessions. Additional data file 9 is a QQ-plot showing the effect of using gDHI to normalize rRHI to reduce the residual effect of sequence difference between targets and probes during mRNA hybridization.

## Supplementary Material

Additional File 1 These probe sets were identified based on the coefficients of variance levels of hybridization indices calculated from genomic DNA hybridization data Click here for file

Additional File 2 A table showing samples used in this study Click here for file

Additional File 3 A table showing correlations between raw and normalized RNA hybridization indices among all 50 samples Click here for file

Additional File 4 (a) One-way and (b) two-way ANOVA tables from analysis of variance Click here for file

Additional File 5 A table showing an example of the Pearson correlation coefficients matrix for a particular gene obtained from 10 pair-wise comparisons among the five accessions Click here for file

Additional File 6 A table showing the sequence variation in promoter regions that alters *cis*-elements Click here for file

Additional File 7 A table showing mRNA expression of genes identified from two-way ANOVA Click here for file

Additional File 8 A figure showing a histogram of coefficient of variance (CV) based on genomic hybridization intensity indices from the five accessions Click here for file

Additional File 9 Two representative samples were shown, the Col-0 4d-seedlings and the NO-0 4d-seedlings before and after genomic DNA normalization. The rest of the 48 samples have similar QQ-profiles Click here for file

## Figures and Tables

**Table 1:** Quantitative RT-PCR confirmation of GeneChip Microarray data for genes 13903_at (At3g54050) and 17392_s_at (At3g53260) in Col-0 and C24

Samples	13903_at			17392_s_at		
	
	log_2_(rRHI)	log_2_(nRHI)	Taqman	log_2_(rRHI)	log_2_(nRHI)	Taqman
Col-0-4 day seedlings	10.11940591	0.911529477	1.348 ± 0.262	10.38351776	0.658285681	0.362 ± 0.024
Col-0-2 week leaf	11.80337083	2.595494397	4.652 ± 0.389	10.56878747	0.84355539	0.299 ± 0.050
Col-0-11 week leaf	10.77324577	1.565369327	1.415 ± 0.336	10.33789612	0.612664042	0.163 ± 0.052
Col-0-2 week root	7.674725423	-1.533151014	0.134 ± 0.014	11.26384894	1.538616864	1.313 ± 0.324
Col-0-5 week root	7.873250697	-1.334625741	0.590 ± 0.064	10.99787749	1.272645415	0.648 ± 0.246
Col-0-influorescence	10.09145865	0.883582211	1.320 ± 0.247	11.01034472	1.285112643	0.519 ± 0.104
Col-0-flower	10.42134176	1.213465325	2.093 ± 0.658	10.62442631	0.899194238	0.263 ± 0.053
Col-0-young siliques	10.65287316	1.444996723	1.999 ± 2.885	10.57630495	0.851072873	0.430 ± 0.197
Col-0-mature siliques	9.475076913	0.267200476	1.432 ± 2.345	10.80990555	1.084673476	0.473 ± 0.113
C24-4 day seedlings	10.90593269	1.883371001	3.690 ± 0.482	10.20742445	0.596353845	0.321 ± 0.059
C24-2 week leaf	12.29789156	3.275329874	6.819 ± 3.507	10.65702025	1.04594965	0.299 ± 0.044
C24-11 week leaf	12.09006973	3.067508045	6.073 ± 1.283	9.19787898	-0.413191622	0.071 ± 0.037
C24-2 week root	7.550943148	-1.471618541	0.069 ± 0.022	10.89199181	1.280921209	0.790 ± 0.133
C24-5 week root	7.945743693	-1.076817996	0.317 ± 0.087	11.16598953	1.554918929	1.122 ± 0.324
C24-influorescence	10.72350042	1.700938727	2.397 ± 0.304	11.10540542	1.494334819	0.743 ± 0.105
C24-flower	10.71423996	1.691678266	1.054 ± 0.167	9.761854806	0.150784204	0.153 ± 0.048
C24-young siliques	11.01401689	1.991455197	1.885 ± 0.726	10.61478826	1.00371766	0.365 ± 0.058
C24-mature siliques	11.21144986	2.188888168	3.808 ± 0.569	11.24013223	1.629061624	1.002 ± 0.151
Correlation with log_2 _(Taqman assay)	0.925	0.933		0.801	0.821	

gDHI for 13903_at is 591.35 and 520.07 for Col-0 and C24, respectively. gDHI for 17392_s_at is 846.42 and 782.02 for Col-0 and C24, respectively.

**Table 2:** Quantitative RT-PCR confirmation of GeneChip microarray data for genes expressed in Col-0 and L*er *flowers

Probe set ID	Col-flower				L*er*-flower				Fold changes	
	
	rRHI	gDHI	nRHI	Taqman	RHI	gDHI	nRHI	Taqman	L*er*/Col (nRHI)	L*er*/Col (Taqman)
12222_s_at	1407.33	700.57	2.01	0.48 ± 0.16	1440.54	557.60	2.58	0.78 ± 0.13	1.29	1.62
14097_at	610.06	1822.91	0.34	0.13 ± 0.03	899.39	1762.56	0.51	0.70 ± 0.23	1.52	5.56
20561_at	760.62	648.27	1.17	0.90 ± 0.14	625.43	719.12	0.87	0.88 ± 0.24	0.74	0.97
14634_s_at	2914.65	1050.64	2.77	0.31 ± 0.05	4304.12	871.65	4.94	0.88 ± 0.05	1.78	2.85
15290_at	701.80	679.74	1.03	0.35 ± 0.03	965.63	583.78	1.65	1.04 ± 0.06	1.60	2.94
14072_at	2034.34	957.24	1.57	0.85 ± 0.13	2285.99	948.01	1.68	1.08 ± 0.20	1.07	1.27
14172_at	894.36	1042.33	0.86	0.44 ± 0.06	1114.93	1107.46	1.01	0.91 ± 0.08	1.17	2.04
14947_at	1888.06	1250.42	1.51	0.98 ± 0.22	1754.25	981.19	1.79	1.24 ± 0.12	1.18	1.26
16892_at	2688.88	836.69	3.22	0.51 ± 0.05	2798.26	1061.25	2.64	1.10 ± 0.11	0.82	2.17
17860_at	959.84	1263.46	0.76	0.49 ± 0.06	1209.50	1322.29	0.92	1.11 ± 0.13	1.20	2.26
20545_at	2183.17	724.58	3.02	0.59 ± 0.09	1971.40	668.92	2.95	0.99 ± 0.09	0.98	1.686

**Table 3:** Correlation analysis of expression patterns of genes among the five accessions

	Per 1	Per 2	Per 3	Per 4	Per 5	Per 6	Per 7	Per 8	Per 9	Per 10	Average of Per	Observed
10	141	133	127	129	129	130	139	121	130	125	130.4	65
9	263	263	246	268	281	285	271	295	273	259	270.4	271
8	324	324	341	323	336	320	307	324	319	359	327.7	376
7	1555	1555	1542	1539	1499	1508	1541	1524	1521	1505	1528.9	399
6	1523	1523	1575	1505	1515	1547	1557	1495	1524	1539	1530.3	412
5	603	603	590	617	607	629	609	642	632	577	610.9	416
4	692	692	661	725	724	660	668	669	715	719	692.5	471
3	441	441	441	436	440	457	461	457	424	441	443.9	438
2	345	345	375	334	341	351	340	355	357	359	350.2	528
1	360	360	365	382	362	329	345	350	358	350	356.1	600
0	1261	1261	1245	1250	1274	1292	1270	1276	1255	1275	1265.9	3532
	7508	7500	7508	7508	7508	7508	7508	7508	7508	7508		7508

For each gene, the Pearson correlation coefficient was calculated for all the 10 pairwise comparisons among the five accessions, as described in Materials and methods. Genes were then grouped into 11 groups (0-10) according to the number of comparisons having correlation coefficients less than 0.5 (group 10 corresponds to the genes with *r *< 0.5 from all 10 pairwise comparisons, whereas group 0 corresponds to genes with *r *≥ 0.5 from all 10 pairwise comparisons). These results are given in the Observed column. Columns Per 1 to Per 10 show the numbers of genes from the 10 permuted datasets, as described in Materials and methods. These results are visualized in Figure 2.

**Table 4:** Genes whose expression is different in leaves of the five accessions by one-way ANOVA analysis

Functional category	ATH1 hits	rawp	Bonferroni correction	GenBank ID	Description
01 METABOLISM					
17946_s_at	At1g03410	2.84132E-06	0.0213326	gb|AAB97721.1|	2-Oxoglutarate-dependent dioxygenase, putative
19689_at	At5g24140	7.0739E-07	0.0053111	emb|CAA06771.1|	Squalene monooxygenase 2 (squalene epoxidase 2) (SQP2) (SE2)
12277_at	At1g47600	6.47203E-07	0.0048592	gb|AAD46026.1|	Glycosyl hydrolase family 1, similar to thioglucosidase
18836_at	At2g24710	5.09671E-06	0.0382661	gb|AAD26894.1|	Plant glutamate receptor family (GLR2.3)
17620_s_at	At2g42990	3.79611E-06	0.0285012	gb|AAD21711.1|	GDSL-motif lipase/hydrolase protein similar to family II lipase EXL3
20514_i_at	At2g15370	1.35384E-08	0.0001016	gb|AAD22287.1|	Similar to xyloglucan fucosyltransferase
					
02 ENERGY					
12277_at	At1g47600	6.47203E-07	0.0048592	gb|AAD46026.1|	Glycosyl hydrolase family 1, similar to thioglucosidase
					
10 CELL CYCLE AND DNA PROCESSING					
18830_at	At2g32790	1.27302E-06	0.0095579	gb|AAC04484.1|	Ubiquitin-conjugating enzyme
15785_g_at	At1g08840	2.79162E-06	0.0209595	gb|AAB70418.1|	Hypothetical protein gene overlaps Sp6 end of F7G19
					
11 TRANSCRIPTION					
12869_s_at	At4g11880	3.45683E-06	0.0259539	gb|AAC49082.1|	MADS-box protein AGL14
16072_s_at	At5g65790	2.97265E-06	0.0223187	gb|AAC83623.1|	Identical to putative transcription factor (MYB68)
13575_at	At4g03430	6.50883E-06	0.0488683	gb|AAD11585.1|	Similar to yeast pre-mRNA splicing factors
20254_at	At2g22390	2.41214E-06	0.0181104	gb|AAD22360.1|	
12366_s_at	At4g11880	2.5787E-06	0.0193608	emb|CAB44326.1|	MADS-box protein AGL14
14885_at	At4g21340	2.22259E-06	0.0166872	emb|CAA20199.1|	Expressed protein, putative bHLH transcription factor (bHLH103)
18830_at	At2g32790	1.27302E-06	0.0095579	gb|AAC04484.1|	Ubiquitin-conjugating enzyme
19244_s_at	At2g04230	2.84687E-06	0.0213743	gb|AAD27915.1|	F-box protein family, contains F-box domain
19279_i_at	At4g21040	7.07742E-07	0.0053137	emb|CAB45899.1|	Dof zinc finger protein, finger protein rolB
13306_at	At2g41070	4.38217E-06	0.0329013	gb|AAD12004.1|	bZIP family transcription factor, contains a bZIP transcription factor basic domain signature
13343_at	At1g34310	4.60448E-08	0.0003457	gb|AAD39615.1|	Transcriptional factor B3 family protein / auxin-responsive factor AUX/IAA-related
15224_at	At1g61540	8.21522E-08	0.0006168	gb|AAD25554.1|	Kelch repeat containing F-box protein family low similarity to SKP1 interacting partner 6
15227_at	At2g01280	5.89076E-06	0.0442278	gb|AAD14528.1|	Transcription factor -related, putative transcription factor IIIB 70 KD subunit (TFIIIB)
16263_at	At2g02320	3.12005E-06	0.0234253	gb|AAC78515.1|	F-box protein (SKP1 interacting partner 3-related)
17145_at	At1g10110	6.9462E-08	0.0005215	gb|AAC34337.1|	Contains Pfam PF00646: F-box domain; similar to F-box protein family, AtFBX7
13863_at	At2g21470	9.36899E-07	0.0070342	gb|AAD23691.1|	Nearly identical to SUMO activating enzyme 2 (SAE2)
12599_at	At2g29910	2.33543E-10	0.0000018	gb|AAD23631.1|	F-box protein family contains F-box domain Pfam:PF00646
12913_at	At4g32880	1.90072E-06	0.0142706	emb|CAA90703.1|	Identical to HD-zip transcription factor (athb-8)
13216_s_at	At1g26310	8.05827E-07	0.0060501	gb|AAA64789.1|	Floral regulatory gene CAULIFLOWER
12863_r_at	At4g18960	1.05463E-06	0.0079181	emb|X53579.1|	Floral homeotic protein agamous (AGAMOUS)
					
14 PROTEIN FATE (folding, modification, destination)					
20254_at	At2g22390	2.41214E-06	0.0181104	gb|AAD22360.1|	Pseudogene, putative GTP-binding protein
18830_at	At2g32790	1.27302E-06	0.0095579	gb|AAC04484.1|	Ubiquitin-conjugating enzyme
13863_at	At2g21470	9.36899E-07	0.0070342	gb|AAD23691.1|	Nearly identical to SUMO activating enzyme 2 (SAE2)
					
16 PROTEIN WITH BINDING FUNCTION OR COFACTOR REQUIREMENT (structural or catalytic)					
20254_at	At2g22390	2.41214E-06	0.0181104	gb|AAD22360.1|	
18836_at	At2g24710	5.09671E-06	0.0382661	gb|AAD26894.1|	Plant glutamate receptor family (GLR2.3)
16262_at	At2g46850	4.75288E-06	0.0356846	gb|AAC34215.2|	Ser/Thr protein kinase -related
					
20 CELLULAR TRANSPORT, TRANSPORT FACILITATION AND TRANSPORT ROUTES					
20254_at	At2g22390	2.41214E-06	0.0181104	gb|AAD22360.1|	Pseudogene, putative GTP-binding protein
18830_at	At2g32790	1.27302E-06	0.0095579	gb|AAC04484.1|	Ubiquitin-conjugating enzyme
18836_at	At2g24710	5.09671E-06	0.0382661	gb|AAD26894.1|	Plant glutamate receptor family (GLR2.3)
17618_at	At2g31910	3.44193E-06	0.0258420	gb|AAD32281.1|	Similar to monovalent cation:proton antiporter family 2
					
30 CELLULAR COMMUNICATION/SIGNAL TRANSDUCTION MECHANISM					
20254_at	At2g22390	2.41214E-06	0.0181104	gb|AAD22360.1|	Pseudogene, putative GTP-binding protein
16816_at	At1g19230	5.65137E-06	0.0424305	gb|AAC39478.1|	Respiratory burst oxidase protein E (NADPH oxidase) (RbohE)
18836_at	At2g24710	5.09671E-06	0.0382661	gb|AAD26894.1|	Plant glutamate receptor family (GLR2.3)
19311_g_at	At2g41210	1.643E-06	0.0123356	gb|AAC78530.2|	Phosphatidylinositol-4-phosphate 5-kinase -related
13343_at	At1g34310	4.60448E-08	0.0003457	gb|AAD39615.1|	Transcriptional factor B3 family protein / auxin-responsive factor AUX/IAA-related
15787_s_at	At1g09090	3.64297E-07	0.0027351	gb|AAB70399.1|	Respiratory burst oxidase protein B (NADPH oxidase) (RbohB)
16262_at	At2g46850	4.75288E-06	0.0356846	gb|AAC34215.2|	Ser/Thr protein kinase -related
					
32 CELL RESCUE, DEFENSE AND VIRULENCE					
20254_at	At2g22390	2.41214E-06	0.0181104	gb|AAD22360.1|	Pseudogene, putative GTP-binding protein
12111_s_at	At4g19240	3.30499E-07	0.0024814	emb|CAA18611.1|	Expressed protein
12258_s_at	At4g14370	6.60533E-06	0.0495928	emb|CAB10216.1|	Disease resistance protein (TIR-NBS-LRR class)
12277_at	At1g47600	6.47203E-07	0.0048592	gb|AAD46026.1|	Glycosyl hydrolase family 1, similar to thioglucosidase
12956_i_at	At1g05170	3.64708E-06	0.0273823	gb|AAB71461.1|	Galactosyltransferase family
16375_at	At1g54480	6.28683E-06	0.0472015	gb|AAD25626.1|	Leucine rich repeat protein family contains leucine rich-repeat (LRR) domains
16816_at	At1g19230	5.65137E-06	0.0424305	gb|AAC39478.1|	Respiratory burst oxidase protein E (NADPH oxidase) (RbohE)
18830_at	At2g32790	1.27302E-06	0.0095579	gb|AAC04484.1|	Ubiquitin-conjugating enzyme
19244_s_at	At2g04230	2.84687E-06	0.0213743	gb|AAD27915.1|	F-box protein family, contains F-box domain
15224_at	At1g61540	8.21522E-08	0.0006168	gb|AAD25554.1|	Kelch repeat containing F-box protein family low similarity to SKP1 interacting partner 6
15787_s_at	At1g09090	3.64297E-07	0.0027351	gb|AAB70399.1|	Respiratory burst oxidase protein B (NADPH oxidase) (RbohB)
16263_at	At2g02320	3.12005E-06	0.0234253	gb|AAC78515.1|	F-box protein (SKP1 interacting partner 3-related)
17145_at	At1g10110	6.9462E-08	0.0005215	gb|AAC34337.1|	Contains Pfam PF00646: F-box domain; similar to F-box protein family, AtFBX7
12599_at	At2g29910	2.33543E-10	0.0000018	gb|AAD23631.1|	F-box protein family contains F-box domain Pfam:PF00646
					
34 INTERACTION WITH THE CELLULAR ENVIRONMENT					
16816_at	At1g19230	5.65137E-06	0.0424305	gb|AAC39478.1|	Respiratory burst oxidase protein E (NADPH oxidase) (RbohE)
18830_at	At2g32790	1.27302E-06	0.0095579	gb|AAC04484.1|	Ubiquitin-conjugating enzyme
15787_s_at	At1g09090	3.64297E-07	0.0027351	gb|AAB70399.1|	Respiratory burst oxidase protein B (NADPH oxidase) (RbohB)
					
36 INTERACTION WITH THE ENVIRONMENT (systemic)					
17946_s_at	At1g03410	2.84132E-06	0.0213326	gb|AAB97721.1|	2-Oxoglutarate-dependent dioxygenase, putative
					
38 TRANSPOSABLE ELEMENTS, VIRAL AND PLASMID PROTEINS					
16731_at	At2g11690	1.06284E-06	0.0079798	gb|AAD28679.1|	Pseudogene
18340_at	At4g07700	2.79501E-06	0.0209849	gb|AAD29786.1|	Athila transposon protein -related
					
40 CELL FATE					
18830_at	At2g32790	1.27302E-06	0.0095579	gb|AAC04484.1|	Ubiquitin-conjugating enzyme
					
41 DEVELOPMENT (systemic)					
17677_at	At1g03910	1.54447E-06	0.0115959	gb|AAD10685.1|	Hypothetical protein
13216_s_at	At1g26310	8.05827E-07	0.0060501	gb|AAA64789.1|	Floral regulatory gene CAULIFLOWER
12863_r_at	At4g18960	1.05463E-06	0.0079181	emb|X53579.1|	Floral homeotic protein agamous (AGAMOUS)
					
42 BIOGENESIS OF CELLULAR COMPONENTS					
20254_at	At2g22390	2.41214E-06	0.0181104	gb|AAD22360.1|	Pseudogene, putative GTP-binding protein
18830_at	At2g32790	1.27302E-06	0.0095579	gb|AAC04484.1|	Ubiquitin-conjugating enzyme
13343_at	At1g34310	4.60448E-08	0.0003457	gb|AAD39615.1|	Transcriptional factor B3 family protein / auxin-responsive factor AUX/IAA-related
					
43 CELL TYPE DIFFERENTIATION					
20254_at	At2g22390	2.41214E-06	0.0181104	gb|AAD22360.1|	Pseudogene, putative GTP-binding protein
18830_at	At2g32790	1.27302E-06	0.0095579	gb|AAC04484.1|	Ubiquitin-conjugating enzyme
					
70 SUBCELLULAR LOCALIZATION					
19689_at	At5g24140	7.0739E-07	0.0053111	emb|CAA06771.1|	Squalene monooxygenase 2 (squalene epoxidase 2) (SQP2) (SE2)
20254_at	At2g22390	2.41214E-06	0.0181104	gb|AAD22360.1|	Pseudogene, putative GTP-binding protein
18830_at	At2g32790	1.27302E-06	0.0095579	gb|AAC04484.1|	Ubiquitin-conjugating enzyme
18836_at	At2g24710	5.09671E-06	0.0382661	gb|AAD26894.1|	Plant glutamate receptor family (GLR2.3)
13343_at	At1g34310	4.60448E-08	0.0003457	gb|AAD39615.1|	Transcriptional factor B3 family protein / auxin-responsive factor AUX/IAA-related
13863_at	At2g21470	9.36899E-07	0.0070342	gb|AAD23691.1|	Nearly identical to SUMO activating enzyme 2 (SAE2)
15785_g_at	At1g08840	2.79162E-06	0.0209595	gb|AAB70418.1|	Hypothetical protein gene overlaps Sp6 end of F7G19
13216_s_at	At1g26310	8.05827E-07	0.0060501	gb|AAA64789.1|	Floral regulatory gene CAULIFLOWER
14356_at	At5g59370	3.6446E-08	0.0002736	gb|AAB39403.1|	Identical to SP|P53494 Actin 4
12863_r_at	At4g18960	1.05463E-06	0.0079181	emb|X53579.1|	Floral homeotic protein agamous (AGAMOUS)
					
No hits to TIGR gene prediction					
20512_at	4.2379E-07	0.0031818	gb|AC002336.3|	*Arabidopsis thaliana *chromosome 2 clone T2P4 map CIC10A06, complete	
18049_s_at	5.37206E-07	0.0040333	emb|AJ132404.1|	*Arabidopsis thaliana *anti-sense transcript, AKL kinase-like gene	

**Table 5:** Genes whose expression is affected by accession-by-organ interaction, identified through two-way ANOVA analysis

Functional category	ATH1 hits	Pr(F)-accessions	Pr(F)-Organs	Pr(F)-accessions: organs	Bonferroni corrected Pr(F)-accessions: organs	Description
41 DEVELOPMENT (systemic)						
18715_at	At1g14930	1.0285E-05	1.3523E-13	1.2253E-08	9.1998E-05	Major latex protein (MLP)-related low similarity to major latex protein
18229_at	At1g14940	5.4018E-07	3.5527E-15	3.3317E-10	2.5014E-06	Major latex protein (MLP)-related low similarity to major latex protein
18717_at	At1g14950	8.6683E-04	2.3537E-14	3.8173E-07	2.8661E-03	Major latex protein (MLP)-related low similarity to major latex protein
17893_at	At2g23110	2.2913E-06	1.7710E-10	3.0508E-07	2.2906E-03	Late embryogenesis abundant proteins -related
12731_f_at	At2g26960	1.1209E-09	4.2244E-09	1.6659E-09	1.2507E-05	MYB family transcription factor
20004_s_at	At2g35300	3.0731E-06	1.2479E-13	1.4412E-06	1.0820E-02	Late embryogenesis abundant proteins -related identical to GB:X91917
13674_s_at	At2g36640	9.1097E-07	1.4619E-11	8.2287E-07	6.1781E-03	Nearly identical to LEA protein in group 3
17038_s_at	At2g36640	6.4830E-06	1.4944E-10	5.8337E-06	4.3799E-02	Nearly identical to LEA protein in group 3
16896_s_at	At2g41260	3.9707E-11	1.1213E-14	3.9237E-10	2.9459E-06	Glycine-rich, identical to late-embryogenesis abundant M17 protein GI:3342551
19355_s_at	At2g41280	3.7790E-09	6.5988E-10	3.4337E-07	2.5780E-03	Late embryogenesis abundant M10 protein identical to GB:AF076979
15747_at	At2g42560	5.1854E-08	6.4206E-12	3.2085E-07	2.4090E-03	Late embryogenesis abundant (LEA) domain-containing protein
15604_s_at	At3g15400	4.6444E-07	4.5835E-09	3.5477E-06	2.6636E-02	Identical to anther development protein ATA20 GB:AAC50042
19918_at	At3g15670	3.7835E-08	1.5210E-14	4.4004E-08	3.3038E-04	Similar to SP|P13934 Late embryogenesis abundant protein 76 (LEA 76)
18872_at	At3g17520	4.2978E-10	1.1102E-16	2.5048E-09	1.8806E-05	Low similarity to PIR|S04045|S04045 embryonic abundant protein D-29
17282_s_at	At3g51810	1.4069E-08	1.5599E-13	6.4057E-10	4.8094E-06	Embryonic abundant protein AtEm1
20682_g_at	At4g26740	6.4621E-04	2.8866E-15	1.0571E-06	7.9364E-03	Embryo-specific protein 1 (ATS1) putative Ca2+-binding EF-hand protein
13675_s_at	At3g22500	8.1351E-08	7.9450E-09	7.7592E-07	5.8256E-03	LEA protein, putative
					
04 STORAGE PROTEIN					
18295_s_at	At1g03880	3.5027E-08	0.0000E+00	1.9892E-10	1.4935E-06	12S seed storage protein (CRB)
13200_s_at	At1g03880	2.0300E-05	2.4425E-15	1.7983E-07	1.3502E-03	12S seed storage protein (CRB)
20221_at	At1g03890	8.7822E-06	5.1070E-15	2.5720E-07	1.9311E-03	Globulin (seed storage protein) family similar to *Arabidopsis thaliana *12S seed storage proteins SP|P15455
20222_g_at	At1g03890	2.3617E-05	2.7756E-15	2.5729E-07	1.9317E-03	globulin (seed storage protein) family similar to *Arabidopsis thaliana *12S seed storage proteins SP|P15455
20535_s_at	At2g28490	2.4914E-03	1.1269E-13	3.8367E-06	2.8806E-02	Cupin domain-containing protein similar to preproMP27-MP32 [Cucurbita cv. Kurokawa Amakuri]
15983_s_at	At4g27140	3.1858E-04	1.4433E-15	2.6036E-07	1.9547E-03	2S seed storage protein 1 (NWMU1 - 2S albumin 1) identical to SP|P15457
15984_s_at	At4g27170	8.4937E-06	0.0000E+00	6.1932E-09	4.6498E-05	2S seed storage protein 4 (NWMU2-2S albumin 4) identical to SP|P15460
13449_at	At4g36700	1.5016E-05	2.9865E-14	3.3621E-06	2.5242E-02	Cupin domain-containing protein low similarity to preproMP27-MP32 from Cucurbita cv. Kurokawa Amakuri
16025_s_at	At4g28520	6.5162E-09	0.0000E+00	2.2615E-10	1.6980E-06	12S seed storage protein (cruciferin), putative
16425_s_at	At5g44120	2.4424E-08	6.1062E-15	3.4512E-07	2.5912E-03	12S seed storage protein (CRA1)
13201_at	At5g54740	3.4456E-08	0.0000E+00	1.8704E-11	1.4043E-07	2S seed storage protein family protein
13194_at	At4g27160	1.0828E-06	5.7732E-15	2.4480E-07	1.8380E-03	NWMU3 - 2S albumin 3 precursor, seed storage protein AT2S3
13198_i_at	At4g28520	4.1773E-07	4.8295E-14	8.3466E-08	6.2666E-04	12S cruciferin seed storage protein
13199_r_at	At4g28520	9.8653E-08	1.0880E-14	1.8093E-08	1.3585E-04	12S cruciferin seed storage protein
					
32 CELL RESCUE, DEFENSE AND VIRULENCE					
14789_at	At2g15010	1.0120E-04	1.2166E-12	4.2917E-06	3.2222E-02	Similar to thionin [*Arabidopsis thaliana*] gi|1181533|gb|AAC41679
18715_at	At1g14930	1.0285E-05	1.3523E-13	1.2253E-08	9.1998E-05	Low similarity to major latex protein {*Papaver somniferum*}
18229_at	At1g14940	5.4018E-07	3.5527E-15	3.3317E-10	2.5014E-06	Low similarity to major latex protein {*Papaver somniferum*}
18717_at	At1g14950	8.6683E-04	2.3537E-14	3.8173E-07	2.8661E-03	Low similarity to major latex protein {*Papaver somniferum*}
20375_at	At1g48130	2.0800E-05	3.1086E-15	1.2134E-07	9.1102E-04	Peroxiredoxin identical to SP:O04005 from [*Arabidopsis thaliana*]
18716_at	At1g75830	1.0527E-05	4.7479E-10	1.2692E-06	9.5295E-03	Plant defensin protein, putative (PDF1.1)
16450_s_at	At3g50980	1.1415E-05	7.6645E-12	8.6448E-07	6.4905E-03	Dehydrin, putative similar to dehydrin Xero 1
17282_s_at	At3g51810	1.4069E-08	1.5599E-13	6.4057E-10	4.8094E-06	Embryonic abundant protein AtEm1
16892_at	At5g45890	3.2112E-09	0.0000E+00	1.7785E-10	1.3353E-06	Cysteine protease SAG12 identical to senescence-specific protein SAG12
18558_at	At2g21490	3.7353E-07	2.0317E-14	2.1270E-07	1.5969E-03	Putative dehydrin
17310_at	At3g51810	4.4370E-06	4.0301E-14	5.2274E-09	3.9248E-05	Embryonic abundant protein AtEm1
					
01 METABOLISM						
18320_s_at	At1g02790	5.5345E-07	0.0000E+00	1.1637E-07	8.7372E-04	Similar to polygalacturonase
17316_at	At2g16730	8.3049E-09	1.0945E-12	1.0082E-06	7.5697E-03	Glycosyl hydrolase family 35 (beta-galactosidase)
19003_at	At2g25890	1.3232E-05	1.8763E-13	5.8363E-07	4.3819E-03	Oleosin
20375_at	At1g48130	2.0800E-05	3.1086E-15	1.2134E-07	9.1102E-04	Peroxiredoxin identical to SP:O04005 from [*Arabidopsis thaliana*]
18991_s_at	At3g27660	1.6605E-04	3.1308E-14	2.4540E-06	1.8425E-02	Identical to oleosin isoform GB:S71286 from [*Arabidopsis thaliana*]
19435_at	At4g00240	3.9561E-08	2.8820E-06	4.0132E-07	3.0131E-03	Phospholipase D -related
16865_s_at	At3g57510	6.4423E-08	3.7925E-13	6.5117E-06	4.8890E-02	Putative similar to polygalacturonase
20412_s_at	At4g25140	3.9887E-06	4.4409E-16	1.5210E-07	1.1420E-03	Oleosin
12435_s_at	At4g34520	1.3485E-05	1.1102E-16	5.6989E-08	4.2788E-04	Fatty acid elongase 1 (FAE1) identical to fatty acid elongase 1 [GI:881615]
16575_s_at	At5g40420	2.6083E-08	0.0000E+00	7.9331E-09	5.9562E-05	Oleosin
20035_at	At5g44440	1.8623E-07	3.5083E-14	4.1535E-07	3.1184E-03	FAD-linked oxidoreductase family similar to SP|P30986 reticuline oxidase precursor (Berberine-bridge-forming enzyme) (BBE)
					
42 BIOGENESIS OF CELLULAR COMPONENTS					
18320_s_at	At1g02790	5.5345E-07	0.0000E+00	1.1637E-07	8.7372E-04	Similar to polygalacturonase GI:288611 from [Zea mays]
19003_at	At2g25890	1.3232E-05	1.8763E-13	5.8363E-07	4.3819E-03	oleosin
15604_s_at	At3g15400	4.6444E-07	4.5835E-09	3.5477E-06	2.6636E-02	Identical to anther development protein ATA20
18716_at	At1g75830	1.0527E-05	4.7479E-10	1.2692E-06	9.5295E-03	Plant defensin protein, putative (PDF1.1)
18991_s_at	At3g27660	1.6605E-04	3.1308E-14	2.4540E-06	1.8425E-02	Identical to oleosin isoform GB:S71286 from [*Arabidopsis thaliana*]
16865_s_at	At3g57510	6.4423E-08	3.7925E-13	6.5117E-06	4.8890E-02	Similar to polygalacturonase GI:288611 from [*Zea mays*]
13243_r_at	At4g37990	2.8137E-07	4.7398E-09	9.4561E-07	7.0996E-03	Mannitol dehydrogenase (ELI3-2), putative
16575_s_at	At5g40420	2.6083E-08	0.0000E+00	7.9331E-09	5.9562E-05	Oleosin
					
70 SUBCELLULAR LOCALIZATION					
12085_at	At1g04560	7.4897E-04	2.7756E-15	2.3200E-07	1.7418E-03	Expressed protein similar to GB:AAC37469
12731_f_at	At2g26960	1.1209E-09	4.2244E-09	1.6659E-09	1.2507E-05	MYB family transcription factor
17710_at	At2g28340	7.6288E-08	2.4759E-06	6.9175E-07	5.1936E-03	GATA zinc finger protein and genefinder
20375_at	At1g48130	2.0800E-05	3.1086E-15	1.2134E-07	9.1102E-04	Peroxiredoxin identical to SP:O04005 from [*Arabidopsis thaliana*]
16892_at	At5g45890	3.2112E-09	0.0000E+00	1.7785E-10	1.3353E-06	Cysteine protease SAG12 identical to senescence-specific protein SAG12
					
14 PROTEIN FATE (folding, modification, destination)					
14420_at	At2g31980	1.3121E-03	2.8566E-13	3.1987E-06	2.4016E-02	Cysteine proteinase inhibitor B (cystatin B) -related
17282_s_at	At3g51810	1.4069E-08	1.5599E-13	6.4057E-10	4.8094E-06	Embryonic abundant protein AtEm1
20682_g_at	At4g26740	6.4621E-04	2.8866E-15	1.0571E-06	7.9364E-03	Embryo-specific protein 1 (ATS1) putative Ca^2+^-binding EF-hand protein
16892_at	At5g45890	3.2112E-09	0.0000E+00	1.7785E-10	1.3353E-06	Cysteine protease SAG12 identical to senescence-specific protein SAG12
20681_at	At4g26740	1.0968E-05	8.7708E-15	3.7368E-06	2.8056E-02	Embryo-specific protein 1 (ATS1)
17310_at	At3g51810	4.4370E-06	4.0301E-14	5.2274E-09	3.9248E-05	Embryonic abundant protein AtEm1
					
30 CELLULAR COMMUNICATION/SIGNAL TRANSDUCTION MECHANISM					
18958_s_at	At3g15410	1.0215E-06	1.6373E-08	4.1125E-08	3.0876E-04	Leucine rich repeat protein family contains leucine rich-repeat (LRR) domains
19435_at	At4g00240	3.9561E-08	2.8820E-06	4.0132E-07	3.0131E-03	Phospholipase D -related
18958_s_at	At3g15410	1.0215E-06	1.6373E-08	4.1125E-08	3.0876E-04	Leucine rich repeat protein family contains leucine rich-repeat (LRR) domains
20682_g_at	At4g26740	6.4621E-04	2.8866E-15	1.0571E-06	7.9364E-03	Embryo-specific protein 1 (ATS1) putative Ca^2+^-binding EF-hand protein
20681_at	At4g26740	1.0968E-05	8.7708E-15	3.7368E-06	2.8056E-02	Embryo-specific protein 1 (ATS1)
					
11 TRANSCRIPTION					
12731_f_at	At2g26960	1.1209E-09	4.2244E-09	1.6659E-09	1.2507E-05	MYB family transcription factor
17710_at	At2g28340	7.6288E-08	2.4759E-06	6.9175E-07	5.1936E-03	GATA zinc finger protein and genefinder
20375_at	At1g48130	2.0800E-05	3.1086E-15	1.2134E-07	9.1102E-04	Peroxiredoxin identical to SP:O04005 from [*Arabidopsis thaliana*]
16892_at	At5g45890	3.2112E-09	0.0000E+00	1.7785E-10	1.3353E-06	Cysteine protease SAG12 identical to senescence-specific protein SAG12
					
02 ENERGY					
16892_at	At5g45890	3.2112E-09	0.0000E+00	1.7785E-10	1.3353E-06	Cysteine protease SAG12 identical to senescence-specific protein SAG12
					
12 PROTEIN SYNTHESIS					
17871_at	At2g16360	9.2371E-07	9.4722E-08	8.4690E-09	6.3585E-05	40S ribosomal protein S25 (RPS25A)
					
34 INTERACTION WITH THE CELLULAR ENVIRONMENT					
20375_at	At1g48130	2.0800E-05	3.1086E-15	1.2134E-07	9.1102E-04	Peroxiredoxin identical to SP:O04005 from [*Arabidopsis thaliana*]
					
36 INTERACTION WITH THE ENVIRONMENT (systemic)					
20375_at	At1g48130	2.0800E-05	3.1086E-15	1.2134E-07	9.1102E-04	peroxIredoxin identical to SP:O04005 from [*Arabidopsis thaliana*]

**Table 6:** The 65 genes with variable expression patterns among the five accessions

Functional category	ATH1 hits	GenBank ID	Description
30 CELLULAR COMMUNICATION/SIGNAL TRANSDUCTION MECHANISM			
14807_at	At2g17170	gb|AAD25145.1|	Protein kinase family contains protein kinase domain, Pfam:PF00069
12528_at	At2g22200	gb|AAD23620.1|	AP2 domain transcription factor
16848_at	At2g20470	gb|AAD25647.1|	Protein kinase, putative contains protein kinase domain, Pfam:PF00069
15069_s_at	At2g28060	gb|AAC98460.1|	AKINbeta3 protein, protein kinase-related
12358_at	At1g54610	gb|AAC64876.1|	Similar to CRK1 protein GI:7671528 from [*Beta vulgaris*]
18510_at	At1g60630	gb|AAB71975.1|	Leucine rich repeat protein family, similar to receptor kinase GI:498278 from [Petunia integrifolia]
16881_at	At1g69990	gb|AAB61113.1|	Leucine-rich repeat transmembrane protein kinase, putative
18478_at	At1g78530	gb|AAD30583.1|	Protein kinase family contains protein kinase domain, Pfam:PF00069
17223_at	At1g78980	gb|AAC17069.1|	Leucine-rich repeat transmembrane protein kinase, putative
16801_s_at	At4g29990	emb|CAB43834.1|	Identical to light repressible receptor protein kinase
16849_at	At4g36070	emb|CAA18501.1|	Calcium-dependent serine/threonine protein kinase isoform AK1
			
11 TRANSCRIPTION			
18443_at	At2g03060	gb|AAC32924.1|	MADS-box protein
14963_at	At1g09920	gb|AAB60744.1|	Expressed protein, TRAF-type zinc finger-related
19242_at	At2g13570	gb|AAD22680.1|	CCAAT-box binding trancription factor -related
12528_at	At2g22200	gb|AAD23620.1|	AP2 domain transcription factor
12220_at	At2g20100	gb|AAD24387.1|	Expressed protein, bHLH - like protein (bHLH133)
14313_at	At2g26130	gb|AAC31224.1|	Hypothetical protein, zinc finger (C3HC4-type RING finger) family protein
16175_g_at	At2g29610	gb|AAC35234.1|	F-box protein family contains Pfam profile PF00646: F-box domain
14370_at	At1g54550	gb|AAD25633.1|	F-box protein family contains Pfam:PF00646 F-box domain
14760_at	At3g46800	emb|CAB51185.1|	CHP-rich zinc finger protein, putative
16209_s_at	At4g10240	emb|CAB39777.1|	CONSTANS B-box zinc finger family protein
14216_at	At5g01290	gb|AAD56326.1|	mRNA capping enzyme - like protein mRNA capping enzyme (HCE), Homo sapiens
18169_at	At4g31615	emb|CAA19761.1|	Transcriptional factor B3 family low similarity to reproductive meristem gene 1 from [Brassica oleracea var. botrytis]
12282_at	At5g44800	gb|AAC79140.1|	Chromodomain-helicase-DNA-binding (CHD) protein family similar to chromatin remodeling factor CHD3 (PICKLE)
			
20 CELLULAR TRANSPORT, TRANSPORT FACILITATION AND TRANSPORT ROUTES			
20248_at	At2g14670	gb|AAC69375.1|	Sucrose transporter (sucrose-proton symporter), putative
18549_s_at	At2g22950	gb|AAF18608.1|	Potential calcium-transporting ATPase 7, plasma membrane-type
19487_at	At2g25580	gb|AAD31361.1|	Pentatricopeptide (PPR) repeat-containing protein contains Pfam profile PF01535: PPR repeat
17363_s_at	At2g32830	dbj|BAA24280.1|	Identical to inorganic phosphate transporter (PHT5)
17242_at	At2g35540	gb|AAC36167.1|	DnaJ domain-containing protein, contains Pfam profile PF00226: DnaJ domain
12389_at	At1g78720	gb|AAC83037.1|	Protein transport protein sec61 alpha subunit -related
18196_at	At4g14820	emb|CAB10261.1|	Pentatricopeptide (PPR) repeat-containing protein contains Pfam profile PF01535: PPR repeat
19255_at	At4g20770	emb|CAB45843.1|	Pentatricopeptide (PPR) repeat-containing protein contains Pfam profile PF01535: PPR repeat
16748_s_at	At4g21300	emb|CAA17548.1|	Pentatricopeptide (PPR) repeat-containing protein contains INTERPRO:IPR002885 PPR repeats
15355_s_at	At4g21560	emb|CAB36800.1|	Expressed protein hypothetical protein YPL065w yeast, PIR2:S60925
			
70 SUBCELLULAR LOCALIZATION			
18443_at	At2g03060	gb|AAC32924.1|	MADS-box protein
12528_at	At2g22200	gb|AAD23620.1|	AP2 domain transcription factor
12358_at	At1g54610	gb|AAC64876.1|	Similar to CRK1 protein GI:7671528 from [*Beta vulgaris*]
12389_at	At1g78720	gb|AAC83037.1|	Protein transport protein Sec61 alpha subunit -related
15486_at	At4g01880	gb|AAD22650.1|	Expressed protein
12282_at	At5g44800	gb|AAC79140.1|	Chromodomain-helicase-DNA-binding (CHD) protein family similar to chromatin remodeling factor CHD3 (PICKLE)
			
14 PROTEIN FATE (folding, modification, destination)			
19487_at	At2g25580	gb|AAD31361.1|	Pentatricopeptide (PPR) repeat-containing protein contains Pfam profile PF01535: PPR repeat
12655_at	At2g31780	gb|AAD32294.1|	Ariadne protein from Drosophila -related
17242_at	At2g35540	gb|AAC36167.1|	DnaJ domain-containing protein, contains Pfam profile PF00226: DnaJ domain
19797_at	At1g64030	gb|AAC27146.1|	Serpin family similar to phloem serpin-1 [*Cucurbita maxima*] GI:9937311
12389_at	At1g78720	gb|AAC83037.1|	Protein transport protein sec61 alpha subunit -related
18408_s_at	At4g03360	gb|AAD14465.1|	Ubiquitin family contains INTERPRO:IPR000626 ubiquitin domain
18196_at	At4g14820	emb|CAB10261.1|	Pentatricopeptide (PPR) repeat-containing protein contains Pfam profile PF01535: PPR repeat
19255_at	At4g20770	emb|CAB45843.1|	Pentatricopeptide (PPR) repeat-containing protein contains Pfam profile PF01535: PPR repeat
16748_s_at	At4g21300	emb|CAA17548.1|	Pentatricopeptide (PPR) repeat-containing protein contains INTERPRO:IPR002885 PPR repeats
			
32 CELL RESCUE, DEFENSE AND VIRULENCE			
16175_g_at	At2g29610	gb|AAC35234.1|	F-box protein family contains Pfam profile PF00646: F-box domain
17242_at	At2g35540	gb|AAC36167.1|	DnaJ domain-containing protein, contains Pfam profile PF00226: DnaJ domain
14370_at	At1g54550	gb|AAD25633.1|	F-box protein family contains Pfam:PF00646 F-box domain
12358_at	At1g54610	gb|AAC64876.1|	Similar to CRK1 protein GI:7671528 from [*Beta vulgaris*]
16803_at	At1g61230	gb|AAB71472.1|	Jacalin lectin family similar to myrosinase-binding protein homolog
17294_at	At4g19500	emb|CAA16927.2|	Disease resistance protein (TIR-NBS-LRR class), putative
17306_at	At5g35940	gb|AAB63636.1|	Jacalin lectin family similar to myrosinase-binding protein homolog
			
42 BIOGENESIS OF CELLULAR COMPONENTS			
19487_at	At2g25580	gb|AAD31361.1|	Pentatricopeptide (PPR) repeat-containing protein contains Pfam profile PF01535: PPR repeat
20031_at	At4g14310	emb|CAB10210.1|	Expressed protein, peroxisomal membrane protein-related
18196_at	At4g14820	emb|CAB10261.1|	Pentatricopeptide (PPR) repeat-containing protein contains Pfam profile PF01535: PPR repeat
19255_at	At4g20770	emb|CAB45843.1|	Pentatricopeptide (PPR) repeat-containing protein contains Pfam profile PF01535: PPR repeat
16748_s_at	At4g21300	emb|CAA17548.1|	Pentatricopeptide (PPR) repeat-containing protein contains INTERPRO:IPR002885 PPR repeats
17733_at	At4g28090	emb|CAB36778.1|	Pectinesterase (pectin methylesterase), putative, similar to pollen-specific BP10 protein [SP|Q00624] [*Brassica napus*]
17586_at	At5g16850	gb|AAD54777.1|	Telomerase reverse transcriptase
12282_at	At5g44800	gb|AAC79140.1|	Chromodomain-helicase-DNA-binding (CHD) protein family similar to chromatin remodeling factor CHD3 (PICKLE)
			
01 METABOLISM			
17817_at	At2g23096	gb|AAC17826.1|	Oxidoreductase -related temporary gene name assignment
18423_at	At1g51260	gb|AAD30638.1|	Acyl-CoA:1-acylglycerol-3-phosphate acyltransferase, putative
12358_at	At1g54610	gb|AAC64876.1|	Similar to CRK1 protein GI:7671528 from [*Beta vulgaris*]
13726_at	At1g74800	gb|AAD55296.1|	Galactosyltransferase family contains Pfam profile: PF01762 galactosyltransferase
19038_at	At3g52160	emb|CAB41336.1|	Beta-ketoacyl-CoA synthase family protein
17646_at	At4g20080	emb|CAA16616.1|	C2 domain-containing protein contains INTERPRO:IPR000008 C2 domain
14274_at	At5g20980	emb|CAB38313.1|	5-Methyltetrahydropteroyltriglutamate-homocysteine S-methyltransferase - like protein
			
16 PROTEIN WITH BINDING FUNCTION OR COFACTOR REQUIREMENT (structural or catalytic)			
12655_at	At2g31780	gb|AAD32294.1|	Ariadne protein from *Drosophila*-related
18510_at	At1g60630	gb|AAB71975.1|	Leucine rich repeat protein family, similar to receptor kinase GI:498278 from [*Petunia integrifolia*]
16881_at	At1g69990	gb|AAB61113.1|	Leucine-rich repeat transmembrane protein kinase, putative
17223_at	At1g78980	gb|AAC17069.1|	Leucine-rich repeat transmembrane protein kinase, putative
16801_s_at	At4g29990	emb|CAB43834.1|	Identical to light repressible receptor protein kinase
12282_at	At5g44800	gb|AAC79140.1|	Chromodomain-helicase-DNA-binding (CHD) protein family similar to chromatin remodeling factor CHD3 (PICKLE)
			
10 CELL CYCLE AND DNA PROCESSING			
12655_at	At2g31780	gb|AAD32294.1|	Ariadne protein from DROSOPHILA -related
17242_at	At2g35540	gb|AAC36167.1|	DnaJ domain-containing protein, contains Pfam profile PF00226: DnaJ domain
12358_at	At1g54610	gb|AAC64876.1|	Similar to CRK1 protein GI:7671528 from [*Beta vulgaris*]
12282_at	At5g44800	gb|AAC79140.1|	Chromodomain-helicase-DNA-binding (CHD) protein family similar to chromatin remodeling factor CHD3 (PICKLE)
02 ENERGY			
19487_at	At2g25580	gb|AAD31361.1|	Pentatricopeptide (PPR) repeat-containing protein contains Pfam profile PF01535: PPR repeat
18196_at	At4g14820	emb|CAB10261.1|	Pentatricopeptide (PPR) repeat-containing protein contains Pfam profile PF01535: PPR repeat
19255_at	At4g20770	emb|CAB45843.1|	Pentatricopeptide (PPR) repeat-containing protein contains Pfam profile PF01535: PPR repeat
16748_s_at	At4g21300	emb|CAA17548.1|	Pentatricopeptide (PPR) repeat-containing protein contains INTERPRO:IPR002885 PPR repeats
			
38 TRANSPOSABLE ELEMENTS, VIRAL AND PLASMID PROTEINS			
16879_at	At2g05550	gb|AAD24652.1|	non-LTR retroelement reverse transcriptase -related
15400_at	At4g08110	gb|AAD27901.1|	Expressed protein, CACTA-like transposase family (Ptta/En/Spm)
17201_at	At4g13120	emb|CAB41922.1|	Hypothetical protein
			
40 CELL FATE			
12389_at	At1g78720	gb|AAC83037.1|	Protein transport protein sec61 alpha subunit -related
13058_s_at	At4g17580	emb|CAB10538.2|	Similar to SP|Q9LD45 Bax inhibitor-1 (BI-1) (AtBI-1)
			
41 DEVELOPMENT (systemic)			
18443_at	At2g03060	gb|AAC32924.1|	MADS-box protein
12389_at	At1g78720	gb|AAC83037.1|	Protein transport protein sec61 alpha subunit -related
			
34 INTERACTION WITH THE CELLULAR ENVIRONMENT			
12358_at	At1g54610	gb|AAC64876.1|	Similar to CRK1 protein GI:7671528 from [*Beta vulgaris*]
			
36 INTERACTION WITH THE ENVIRONMENT (Systemic)			
12389_at	At1g78720	gb|AAC83037.1|	Protein transport protein sec61 alpha subunit -related
			
12 PROTEIN SYNTHESIS			
16667_at	At3g48960	emb|CAB51060.1|	60S ribosomal protein L13 (RPL13C)

**Table 7:** The combined numbers of polymorphisms and the mutation rates in the promoters, ORFs and exons of seven genes showing high variation in expression

Accession ID/polymophisms	Description	Promoter	ORF	Exon	Promoter	ORF	Exon
		
		Five accessions			All accessions		
At1g28210	Mitochondrial protein (AtJ1), putative	33	23	4	43	32	4
At2g32930	CCCH Zn-finger protein	1	2	0	1	3	1
At2g34290	Putative protein kinase	1	11	11	10	21	21
At3g13445	Transcription initiation factor TFIID-1 (TATA sequence-binding protein 1)	7	2	0	9	3	3
At4g10160	Putative RING Zn-finger protein	7	46	15	16	57	20
At4g39410	WRKY family transcription factor	3	12	0	6	12	6
At2g18790	Phytochrome B (PHYB) photoreceptor	9	10	7	21	82	71
Total number		61	106	37	106	210	126
Rate per kb		8.06	6.11	4.08	14.00	12.10	13.90
