# Supplementary material for: Contribution of transcriptional regulation to natural variations in Arabidopsis
Source: Genome Biol. 2005 Mar 15;6(4):R32. doi: 10.1186/gb-2005-6-4-r32 (PMC1088960; doi:10.1186/gb-2005-6-4-r32)
Supplement: Additional File 6 — A table showing the sequence variation in promoter regions that alters cis-elements [file gb-2005-6-4-r32-S6.doc]

### Supplemental Table VI. Sequence variation in promoters regions that alters cis-elements

| Position from ATG | Accessions with complete elements | Accessions with absent or altered elements | Element in predicted promoters | Details of element |
| --- | --- | --- | --- | --- |
|  |  |  |  |  |
| **At1g28210 mitochondrial protein (AtJ1), putative (Contains small gaps)** | | | | |
| -1083 | Col-0, | Ler, No | CGTCA | ASF1MOTIFCAMV "ASF-1 BINDING SITE" IN CAMV 35S PROMOTER; ASF-1 BINDS TO TWO TGACG MOTIFS; SEE S000023 (AS1); SAME MOTIF IS FOUND IN HBP-1 BINDING SITE OF WHEAT HISTONE H3 GENE; TGACG MOTIFS ARE FOUND IN MANY PROMOTERS AND ARE INVOLVED IN TRANSCRIPTIONAL ACTIVATION OF SEVERAL GENES BY AUXIN AND/OR SALICYLIC ACID; MAY BE RELEVANT TO LIGHT REGULATION; BINDING SITE OF TOBACCO TGA1A (TGA1A-SEQUENCE SPECIFIC BINDING PROTEIN; TGA1A AND B SHOW HOMOLOGY TO CREB; TGA6 IS A NEW MEMBER OF THE TGA FAMILY; |
|  | Col-0, | Ler, No | CGTCA | TGACG-MOTIF | U83904 | HORDEUM VULGARE | CIS-ACTING REGULATORY ELEMENT INVOLVED IN THE MEJA-RESPONSIVENESS |
|  | Ler, No | Col-0, | GTCGG | LTRECOREATCOR15 CORE OF LOW TEMPERATURE RESPONSIVE ELEMENT (LTRE) OF COR15A GENE IN ARABIDOPSIS (A.T.); A PORTION OF REPEAT-C (C-REPEAT), TGGCCGAC, WHICH IS REPEATED TWICE IN COR15A PROMOTER (BAKER ET AL., 1994); ABA RESPONSIVENESS; INVOLVED IN COLD INDUCTION OF BN115 GENE FROM WINTER BRASSICA NAPUS; LTRE; SEE S000157, S000152; LIGHT SIGNALLING MEDIATED BY PHYTOCHROME IS NECESSARY FOR COLD- OR DROUGHT- INDUCED GENE EXPRESSION THROUGH THE C/DRE IN ARABIDOPSIS; SEE S000152; |
|  | Ler, No | Col-0, | CGTCG | CGACGOSAMY3 "CGACG ELEMENT" FOUND IN THE GC-RICH REGIONS OF THE RICE (O.S.) AMY3D AND AMY3E AMYLASE GENES, BUT NOT IN AMY3E GENE; MAY FUNCTION AS A COUPLING ELEMENT FOR THE G BOX ELEMENT; |
|  | Col-0-0, | Ler, No | CGTCA | CGTCA-MOTIF | U83904 | HORDEUM VULGARE | CIS-ACTING REGULATORY ELEMENT INVOLVED IN THE MEJA-RESPONSIVENESS |
| -1056 | Col-0, No | Ler, WS | ATTGG | CAAT-BOX | L41253 | LYCOPERSICON ESCULENTUM | COMMON CIS-ACTING ELEMENT IN PROMOTER AND ENHANCER REGIONS |
|  | Col-0, No | Ler, WS | ATTGG | CAAT-BOX | Y13535 | BRASSICA OLERACEA | COMMON CIS-ACTING ELEMENT IN PROMOTER AND ENHANCER REGIONS |
|  | Col-0, No | Ler, WS | ATTGG | CAAT-BOX | D45890 | ORYZA SATIVA | COMMON CIS-ACTING ELEMENT IN PROMOTER AND ENHANCER REGIONS |
|  | Col-0, No | Ler, WS | ATTGG | CAAT-BOX | X98521 | BRASSICA OLERACEA | COMMON CIS-ACTING ELEMENT IN PROMOTER AND ENHANCER REGIONS |
|  | Col-0, No | Ler, WS | ATTGG | CAAT-BOX | Z13987 | SOLANUM TUBEROSUM | COMMON CIS-ACTING ELEMENT IN PROMOTER AND ENHANCER REGIONS |
|  | Col-0, No | Ler, WS | ATTGG | CAAT-BOX | U45858 | ZEA MAYS | COMMON CIS-ACTING ELEMENT IN PROMOTER AND ENHANCER REGIONS |
|  | Col-0, No | Ler, WS | ATTGG | CAAT-BOX | Z35160 | SOLANUM TUBEROSUM | COMMON CIS-ACTING ELEMENT IN PROMOTER AND ENHANCER REGIONS |
|  | Col-0, No | Ler, WS | ATTGG | CAAT-BOX | D13044 | ARABIDOPSIS THALIANA | COMMON CIS-ACTING ELEMENT IN PROMOTER AND ENHANCER REGIONS |
|  | Col-0, No | Ler, WS | GTGA | GTGANTG10 "GTGA MOTIF" FOUND IN THE PROMOTER OF THE TABACCO (N.T.) LATE POLLEN GENE G10 WHICH SHOWS HOMOLOGY TO PECTATE LYASE AND IS THE PUTATIVE HOMOLOGUE OF THE TOMATO GENE LAT56; LOCATED BETWEEN -96 AND -93; SEE S000280; |
|  | Col-0, No | Ler, WS | ATTGG | CCAATBOX1 COMMON SEQUENCE FOUND IN THE 5'-NON-CODING REGIONS OF EUKARYOTIC GENES; |
| -1042 | No-0 | all others (no data for C24, Bs-1, Tsu-0). | AAAG | DOFCOREZM CORE SITE REQUIRED FOR BINDING OF DOF PROTEINS IN MAIZE (Z.M.); DOF PROTEINS ARE DNA BINDING PROTEINS, WITH PRESUMABLY ONLY ONE ZINC FINGER, AND ARE UNIQUE TO PLANTS; FOUR CDNAS ENCODING DOF PROTEINS, DOF1, DOF2, DOF3 AND PBF, HAVE BEEN ISOLATED FROM MAIZE; PBF IS AN ENDOSPERM SPECIFIC DOF PROTEIN THAT BINDS TO PROLAMIN BOX; MAIZE DOF1 ENHANCES TRANSCRIPTION FROM THE PROMOTERS OF BOTH CYTOSOLIC ORTHOPHOSPHATE KINASE (CYPPDK) AND A NON-PHOTOSYNTHETIC PEPC GENE; MAIZE DOF2 SUPRESSED THE C4PEPC PROMOTER; |
|  | No-0 | all others (no data for C24, Bs-1, Tsu-0). | TAAAA | TATA-BOX | L41253 | LYCOPERSICON ESCULENTUM | CORE PROMOTER ELEMENT AROUND -30 OF TRANSCRIPTION START |
| -1012 | all others (no data for C24). | Col-0. | ATTG | CAAT-BOX | X78205 | HORDEUM VULGARE | COMMON CIS-ACTING ELEMENT IN PROMOTER AND ENHANCER REGIONS |
|  | all others (no data for C24). | Col-0. | TTTATT | POLASIG1 "POLYA SIGNAL"; POLY A SIGNAL FOUND IN LEGA GENE OF PEA, RICE ALPHA-AMYLASE; -10 TO -30 IN THE CASE OF ANIMAL GENES. |
|  | all others (no data for C24). | Col-0. | ATTTTTA | SEF4MOTIFGM7S "SEF4 BINDING SITE"; SOYBEAN (G.M.) CONSENSUS SEQUENCE FOUND IN 5'UPSTREAM REGION (-199) OF BETA-CONGLYCININ (7S GLOBULIN) GENE (GMG17.1); "BINDING WITH SEF4 (SOYBEAN EMBRYO FACTOR 4)"; R=A/G; |
|  | all others (no data for C24). | Col-0. | AATATTTTTATT | AT1BOX "AT-1 BOX (AT-RICH ELEMENT)" FOUND IN THE PROMOTER REGION OF THE GENES FOR TOBACCO ( N.P.) CHLOROPHYLL A/B BINDING PROTEIN (CAB) AND SMALL SUBUNIT OF RIBULOSE-1,5-BISPHOSPHATE CARBOXYLASE (RBCS); DELETION OF A REGION CONTAINING THE AT-1 SITE IN THE TOMATO RBCS3A GENE STRONGLY INHIBITED REPORTER GENE EXPRESSION, WHEREAS AT-1 SITE IN N. PLUMBAGINIFOLIA CAB GENE (CAB-E) IS IN A NEGATIVE ELEMENT (TERZAGHI & CASHMORE, 1995); |
|  | all others (no data for C24). | Col-0. | ATTG | CAAT-BOX | S44160 | LYCOPERSICON ESCULENTUM | COMMON CIS-ACTING ELEMENT IN PROMOTER AND ENHANCER REGIONS |
|  | all others (no data for C24). | Col-0. | ATTG | CAATBOX1 "CAAT PROMOTER CONSENSUS SEQUENCE" FOUND IN LEGA GENE OF PEA; |
|  | all others (no data for C24). | Col-0. | ATTG | CAAT-BOX | L02124 | NICOTIANA TABACUM | COMMON CIS-ACTING ELEMENT IN PROMOTER AND ENHANCER REGIONS |
| -993 | all others (no data for C24). | No | AGAAACTT | AE-BOX | L14749 | ARABIDOPSIS THALIANA | PART OF A MODULE FOR LIGHT RESPONSE |
|  | No | all others (no data for C24). | GAAAAA | GT1CONSENSUS CONSENSUS GT-1 BINDING SITE IN MANY LIGHT-REGULATED GENES, E.G., RBCS FROM MANY SPECIES, PHYA FROM OAT AND RICE, SPINACH RCA AND PETA, AND BEAN CHS15; R=A/G; W=A/T; FOR A COMPILATION OF RELATED GT ELEMENTS AND FACTORS, SEE VILLAIN ET AL. (1996); GT-1 CAN STABILIZE THE TFIIA-TBP-DNA (TATA BOX) COMPLEX; THE ACTIVATION MECHANISM OF GT-1 MAY BE ACHIEVED THROUGH DIRECT INTERACTION BETWEEN TFIIA AND GT-1; BINDING OF GT-1-LIKE FACTORS TO THE PR-1A PROMOTER INFLUENCES THE LEVEL OF SA-INDUCIBLE GENE EXPRESSION; |
| -959 | all others (no data for C24). | Col-0 | GATA | GATABOX "GATA BOX"; GATA MOTIF IN CAMV 35S PROMOTER; BINDING WITH ASF-2; THREE GATA BOX REPEATS WERE FOUND IN THE PROMOTER OF PETUNIA (P.H.) CHLOROPHYLL A/B BINDING PROTEIN, CAB22 GENE; REQUIRED FOR HIGH LEVEL, LIGHT REGULATED, AND TISSUE SPECIFIC EXPRESSION; CONSERVED IN THE PROMOTER OF ALL LHCII TYPE I CAB GENES; |
| -956 | Es-0 | all others (no data for C24). | TATC | GATABOX "GATA BOX"; GATA MOTIF IN CAMV 35S PROMOTER; BINDING WITH ASF-2; THREE GATA BOX REPEATS WERE FOUND IN THE PROMOTER OF PETUNIA (P.H.) CHLOROPHYLL A/B BINDING PROTEIN, CAB22 GENE; REQUIRED FOR HIGH LEVEL, LIGHT REGULATED, AND TISSUE SPECIFIC EXPRESSION; CONSERVED IN THE PROMOTER OF ALL LHCII TYPE I CAB GENES; |
| -869 | Col-0, No | Ler, WS | TATC | GATABOX "GATA BOX"; GATA MOTIF IN CAMV 35S PROMOTER; BINDING WITH ASF-2; THREE GATA BOX REPEATS WERE FOUND IN THE PROMOTER OF PETUNIA (P.H.) CHLOROPHYLL A/B BINDING PROTEIN, CAB22 GENE; REQUIRED FOR HIGH LEVEL, LIGHT REGULATED, AND TISSUE SPECIFIC EXPRESSION; CONSERVED IN THE PROMOTER OF ALL LHCII TYPE I CAB GENES; |
|  | Col-0, No | Ler, WS | CAAACATATC | CIACADIANLELHC REGION NECESSARY FOR CIRCADIAN EXPRESSION OF TOMATO (L.E.) LHC GENE; |
|  | Col-0, No | Ler, WS | CAAACATATC | CIRCADIAN CLOCK | | LYCOPERSICON ESCULENTUM | CIS-ACTING REGULATORY ELEMENT INVOLVED IN CIRCADIAN CONTROL |
|  | Col-0, No | Ler, WS | CAATTATTA | ATHB6COREAT CONSENSUS BINDING SEQUENCE FOR ARABIDOPSIS (A.T.) HOMEODOMAIN-LEUCINE ZIPPER PROTEIN, ATHB6; ATHB6 IS A TARGET OF THE PROTEIN PHOSPHATASE ABI1 AND REGULATES HORMONE RESPONSES; SEE S000371; |
|  | Ler, WS | Col-0, No | ATTAAT | BOX 4 | X15473 | PETROSELINUM CRISPUM | PART OF A CONSERVED DNA MODULE INVOLVED IN LIGHT RESPONSIVENESS |
|  | Ler, WS | Col-0, No | TAATTATTA | HDZIP2ATATHB2 BINDING SITE OF THE ARABIDOPSIS (A.T.) HOMEOBOX GENE (ATHB-2) FOUND IN ITS OWN PROMOTER; LOCATED BETWEEN -72 AND -80; SIMILAR TO THE HD-ZIP-2 BINDING CONSENSUS SEQUENCE; ATHB-2 IS REGULATED BY LIGHT SIGNALS WHICH FUNCTION AS A NEGATIVE AUTOREGULATOR OF ITS OWN GENE; M=C/A; |
|  | Ler, WS | Col-0, No | TATTAAT | TATABOX3 "TATA BOX"; TATA BOX FOUND IN THE 5'UPSTREAM REGION OF SWEET POTATO SPORAMIN A GENE; |
|  | Col-0, , No | Ler, WS | CAATT | CAAT-BOX | Z26331 | GLYCINE MAX | COMMON CIS-ACTING ELEMENT IN PROMOTER AND ENHANCER REGIONS |
| -853 | all others (no data for C24). | Col-0. | CAAAT | CAAT-BOX | Y13108 | BRASSICA RAPA | COMMON CIS-ACTING ELEMENT IN PROMOTER AND ENHANCER REGIONS |
|  | all others (no data for C24). | Col-0. | CAAAT | CAAT-BOX | D10661 | PISUM SATIVUM | COMMON CIS-ACTING ELEMENT IN PROMOTER AND ENHANCER REGIONS |
| -749 | all others (no data for C24). | Col-0. | TATATA | TATA-BOX | X61937 | BRASSICA NAPUS | CORE PROMOTER ELEMENT AROUND -30 OF TRANSCRIPTION START |
|  | Col-0 | all others (no data for C24). | CAAT | CAAT-BOX | X78205 | HORDEUM VULGARE | COMMON CIS-ACTING ELEMENT IN PROMOTER AND ENHANCER REGIONS |
|  | all others (no data for C24). | Col-0. | TATATA | TATA-BOX | X16184 | GLYCINE MAX | CORE PROMOTER ELEMENT AROUND -30 OF TRANSCRIPTION START |
|  | all others (no data for C24). | Col-0. | TATAAAT | TATABOX2 "TATA BOX"; TATA BOX FOUND IN THE 5'UPSTREAM REGION OF PEA LEGA GENE; SPORAMIN A OF SWEET POTATO; |
|  | all others (no data for C24). | Col-0. | TATATA | TATA-BOX | Z35160 | SOLANUM TUBEROSUM | CORE PROMOTER ELEMENT AROUND -30 OF TRANSCRIPTION START |
|  | all others (no data for C24). | Col-0. | TATAAAT | TATA-BOX | X14597 | PETUNIA HYBRIDA | CORE PROMOTER ELEMENT AROUND -30 OF TRANSCRIPTION START |
|  | Col-0 | all others (no data for C24). | TATC | GATABOX "GATA BOX"; GATA MOTIF IN CAMV 35S PROMOTER; BINDING WITH ASF-2; THREE GATA BOX REPEATS WERE FOUND IN THE PROMOTER OF PETUNIA (P.H.) CHLOROPHYLL A/B BINDING PROTEIN, CAB22 GENE; REQUIRED FOR HIGH LEVEL, LIGHT REGULATED, AND TISSUE SPECIFIC EXPRESSION; CONSERVED IN THE PROMOTER OF ALL LHCII TYPE I CAB GENES; |
|  | all others (no data for C24). | Col-0. | TATATAAA | TATAPVTRNALEU "TATA-LIKE MOTIF"; A TATA-LIKE SEQUENCE FOUND IN PHASEOLUS VULGARIS TRNALEU GENE PROMOTER; FREQUENTLY OBSERVED UPSTREAM OF PLANT TRNA GENES; FOUND IN MAIZE GLYCOLYTIC GLYCERALDEHYDE-3-PHOSPATE DEHYDROGENASE 4 (GAPC4) GENE PROMOTER; BINDING SITE OF TATA BINDING PROTEIN (TBP); |
|  | all others (no data for C24). | Col-0. | TATATA | TATA-BOX | AJ224116 | HELIANTHUS ANNUUS | CORE PROMOTER ELEMENT AROUND -30 OF TRANSCRIPTION START |
|  | all others (no data for C24). | Col-0. | ATATATA | TATA-BOX | X05979 | PISUM SATIVUM | CORE PROMOTER ELEMENT AROUND -30 OF TRANSCRIPTION START |
|  | all others (no data for C24). | Col-0. | TATAAAT | TATA-BOX | Y13108 | BRASSICA RAPA | CORE PROMOTER ELEMENT AROUND -30 OF TRANSCRIPTION START |
|  | all others (no data for C24). | Col-0. | TATAAATT | TATA-BOX | X03710 | ANTIRRHINUM MAJUS | CORE PROMOTER ELEMENT AROUND -30 OF TRANSCRIPTION START |
|  | Col-0, | all others (no data for C24). | CAAT | CAAT-BOX | S44160 | LYCOPERSICON ESCULENTUM | COMMON CIS-ACTING ELEMENT IN PROMOTER AND ENHANCER REGIONS |
|  | all others (no data for C24). | Col-0. | TATAAAT | TATA-BOX | AC | AC | CORE PROMOTER ELEMENT AROUND -30 OF TRANSCRIPTION START |
|  | all others (no data for C24). | Col-0. | TATATAA | TATABOX4 "TATA BOX"; TATA BOX FOUND IN THE 5'UPSTREAM REGION OF SWEET POTATO SPORAMIN A GENE; |
|  | all others (no data for C24). | Col-0. | TATAAAT | TATA-BOX | A23331 | ORYZA SATIVA | CORE PROMOTER ELEMENT AROUND -30 OF TRANSCRIPTION START |
|  | all others (no data for C24). | Col-0. | TATAAAT | TATA-BOX | X70333 | BRASSICA OLERACEA | CORE PROMOTER ELEMENT AROUND -30 OF TRANSCRIPTION START |
|  | all others (no data for C24). | Col-0. | TATATA | TATA-BOX | M92354 | ARABIDOPSIS THALIANA | CORE PROMOTER ELEMENT AROUND -30 OF TRANSCRIPTION START |
|  | all others (no data for C24). | Col-0. | TATATA | TATA-BOX | D45890 | ORYZA SATIVA | CORE PROMOTER ELEMENT AROUND -30 OF TRANSCRIPTION START |
|  | all others (no data for C24). | Col-0. | TATATAA | TATA-BOX | AB006777 | ARABIDOPSIS THALIANA | CORE PROMOTER ELEMENT AROUND -30 OF TRANSCRIPTION START |
|  | Col-0, | all others (no data for C24). | CAAT | CAATBOX1 "CAAT PROMOTER CONSENSUS SEQUENCE" FOUND IN LEGA GENE OF PEA; |
|  | all others (no data for C24). | Col-0. | TATATA | TATA-BOX | X16186 | GLYCINE MAX | CORE PROMOTER ELEMENT AROUND -30 OF TRANSCRIPTION START |
|  | all others (no data for C24). | Col-0. | TATAAAT | TATA-BOX | U48862 | PETROSELINUM CRISPUM | CORE PROMOTER ELEMENT AROUND -30 OF TRANSCRIPTION START |
|  | all others (no data for C24). | Col-0. | TATATAA | TATA-BOX | AB006778 | ARABIDOPSIS THALIANA | CORE PROMOTER ELEMENT AROUND -30 OF TRANSCRIPTION START |
|  | all others (no data for C24). | Col-0. | ATATATA | TATA-BOX | U45859 | ZEA MAYS | CORE PROMOTER ELEMENT AROUND -30 OF TRANSCRIPTION START |
|  | Col-0, | all others (no data for C24). | CAAT | CAAT-BOX | L02124 | NICOTIANA TABACUM | COMMON CIS-ACTING ELEMENT IN PROMOTER AND ENHANCER REGIONS |
|  | Col-0, | all others (no data for C24). | CAATT | CAAT-BOX | Z26331 | GLYCINE MAX | COMMON CIS-ACTING ELEMENT IN PROMOTER AND ENHANCER REGIONS |
|  | all others (no data for C24). | Col-0. | TATAAAT | TATA-BOX | U48863 | PETROSELINUM CRISPUM | CORE PROMOTER ELEMENT AROUND -30 OF TRANSCRIPTION START |
|  | all others (no data for C24). | Col-0. | TATATA | TATA-BOX | X16185 | GLYCINE MAX | CORE PROMOTER ELEMENT AROUND -30 OF TRANSCRIPTION START |
|  | all others (no data for C24). | Col-0. | TATATA | TATA-BOX | Z13987 | SOLANUM TUBEROSUM | CORE PROMOTER ELEMENT AROUND -30 OF TRANSCRIPTION START |
|  | all others (no data for C24). | Col-0. | TATAAAT | TATA-BOX | X67833 | BRASSICA JUNCEA | CORE PROMOTER ELEMENT AROUND -30 OF TRANSCRIPTION START |
| -624 | No. | all others (no data for C24). | TATAAT | TATA-BOX | U27107 | BRASSICA NAPUS | CORE PROMOTER ELEMENT AROUND -30 OF TRANSCRIPTION START |
|  | No. | all others (no data for C24). | TATAA | TATA-BOX | D13044 | ARABIDOPSIS THALIANA | CORE PROMOTER ELEMENT AROUND -30 OF TRANSCRIPTION START |
|  | No. | all others (no data for C24). | TATAAT | TATA-BOX | U46217 | PETROSELINUM CRISPUM | CORE PROMOTER ELEMENT AROUND -30 OF TRANSCRIPTION START |
| -601 | Col-0. | all others (no data for C24). | GATA | GATABOX "GATA BOX"; GATA MOTIF IN CAMV 35S PROMOTER; BINDING WITH ASF-2; THREE GATA BOX REPEATS WERE FOUND IN THE PROMOTER OF PETUNIA (P.H.) CHLOROPHYLL A/B BINDING PROTEIN, CAB22 GENE; REQUIRED FOR HIGH LEVEL, LIGHT REGULATED, AND TISSUE SPECIFIC EXPRESSION; CONSERVED IN THE PROMOTER OF ALL LHCII TYPE I CAB GENES; |
|  | Col-0. | all others (no data for C24). | GATAAT | GT1CONSENSUS CONSENSUS GT-1 BINDING SITE IN MANY LIGHT-REGULATED GENES, E.G., RBCS FROM MANY SPECIES, PHYA FROM OAT AND RICE, SPINACH RCA AND PETA, AND BEAN CHS15; R=A/G; W=A/T; FOR A COMPILATION OF RELATED GT ELEMENTS AND FACTORS, SEE VILLAIN ET AL. (1996); GT-1 CAN STABILIZE THE TFIIA-TBP-DNA (TATA BOX) COMPLEX; THE ACTIVATION MECHANISM OF GT-1 MAY BE ACHIEVED THROUGH DIRECT INTERACTION BETWEEN TFIIA AND GT-1; BINDING OF GT-1-LIKE FACTORS TO THE PR-1A PROMOTER INFLUENCES THE LEVEL OF SA-INDUCIBLE GENE EXPRESSION; |
|  | Col-0. | all others (no data for C24). | GATAA | IBOXCORE "I BOX"; "I-BOX"; CONSERVED SEQUENCE UPSTREAM OF LIGHT-REGULATED GENES; CONSERVED SEQUENCE UPSTREAM OF LIGHT-REGULATED GENES OF BOTH MONOCOTS AND DICOTS; SEE IBOX (S000124); |
| -510 | Col-0, C24. | all others | CAAAAAT | SEF4MOTIFGM7S "SEF4 BINDING SITE"; SOYBEAN (G.M.) CONSENSUS SEQUENCE FOUND IN 5'UPSTREAM REGION (-199) OF BETA-CONGLYCININ (7S GLOBULIN) GENE (GMG17.1); "BINDING WITH SEF4 (SOYBEAN EMBRYO FACTOR 4)"; R=A/G; |
|  | C24, Col-0 | all others | AAAAAAAAAA | MARTBOX "T-BOX"; MOTIF FOUND IN SAR (SCAFFOLD ATTACHMENT REGION; OR MATRIX ATTACHMENT REGION, MAR); |
| -275 | No | all others | TGACGT | CELLCYCLE-1B | M25268 | ARABIDOPSIS THALIANA | TRANSACTIVATOR IN THE CELL-CYCLE DEPENDENT TRANSCRIPTION |
|  | No | all others | TGACG | ASF1MOTIFCAMV "ASF-1 BINDING SITE" IN CAMV 35S PROMOTER; ASF-1 BINDS TO TWO TGACG MOTIFS; SEE S000023 (AS1); SAME MOTIF IS FOUND IN HBP-1 BINDING SITE OF WHEAT HISTONE H3 GENE; TGACG MOTIFS ARE FOUND IN MANY PROMOTERS AND ARE INVOLVED IN TRANSCRIPTIONAL ACTIVATION OF SEVERAL GENES BY AUXIN AND/OR SALICYLIC ACID; MAY BE RELEVANT TO LIGHT REGULATION; BINDING SITE OF TOBACCO TGA1A (TGA1A-SEQUENCE SPECIFIC BINDING PROTEIN; TGA1A AND B SHOW HOMOLOGY TO CREB; TGA6 IS A NEW MEMBER OF THE TGA FAMILY; |
|  | No | all others | TGACG | TGACG-MOTIF | U83904 | HORDEUM VULGARE | CIS-ACTING REGULATORY ELEMENT INVOLVED IN THE MEJA-RESPONSIVENESS |
|  | No | all others | TGACGT | HEXMOTIFTAH3H4 "HEXAMER MOTIF" FOUND IN PROMOTER OF WHEAT (T.A.) HISTONE GENES H3 AND H4; CAMV35S; NOS; BINDING WITH HBP-1A AND HBP-1B; BINDING SITE OF WHEAT (T.A.) NUCLEAR PROTEIN HBP-1 (HISTONE DNA BINDING PROTEIN-1); HBP-1 HAS A LEUCINE ZIPPER MOTIF; "HEXAMER MOTIF" IN TYPE 1 ELEMENT MAY PLAY IMPORTANT ROLES IN REGULATION OF REPLICATION- DEPENDENT BUT NOT OF REPLICATION-INDEPENDENT EXPRESSION OF THE WHEAT HISTONE H3 GENE; SEE S000076, S000267 |
|  | No | all others | TTGAC | WBOXATNPR1 "W-BOX" FOUND IN PROMOTER OF ARABIDOPSIS THALIANA (A.T.) NPR1 GENE; LOCATED BETWEEN +70 AND +79 IN TANDEM; THEY WERE RECONGNIZED SPECIFICALLY BY SALICYLIC ACID (SA)-INDUCED WRKY DNA BINDING PROTEINS; SEE S000142 (TTGACC); SEE S000310 (TTTGACT); |
|  | No | all others | TGACG | CGTCA-MOTIF | U83904 | HORDEUM VULGARE | CIS-ACTING REGULATORY ELEMENT INVOLVED IN THE MEJA-RESPONSIVENESS |
|  | No | all others | TGACGT | TGACGTVMAMY "TGACGT MOTIF" FOUND IN THE VIGNA MUNGO (V.M.) ALPHA-AMYLASE (AMY) GENE PROMOTER; LOCATED BETWEEN -128 AND -123; REQUIRED FOR HIGH LEVEL EXPRESSION OF ALPHA-AMYLASE IN THE COTYLEDONS OF THE GERMINATED SEEDS; SEE S000234; |
| -266 | C24, Col-0. | all others. | TATAAAA | TATA-BOX | D88260 | PISUM SATIVUM | CORE PROMOTER ELEMENT AROUND -30 OF TRANSCRIPTION START |
|  | C24, Col-0. | all others. | TATAAAA | TATA-BOX | X95295 | ARABIDOPSIS THALIANA | CORE PROMOTER ELEMENT AROUND -30 OF TRANSCRIPTION START |
|  | C24, Col-0. | all others. | TATAAAA | TATA-BOX | D88261 | PISUM SATIVUM | CORE PROMOTER ELEMENT AROUND -30 OF TRANSCRIPTION START |
|  | C24, Col-0. | all others. | TATAAAA | TATA-BOX | AF060237 | PISUM SATIVUM | CORE PROMOTER ELEMENT AROUND -30 OF TRANSCRIPTION START |
|  | C24, Col-0. | all others. | TATAAAA | TATA-BOX | D88262 | PISUM SATIVUM | CORE PROMOTER ELEMENT AROUND -30 OF TRANSCRIPTION START |
|  | C24, Col-0. | all others. | AATAT | ROOTMOTIFTAPOX1 MOTIF FOUND BOTH IN PROMOTERS OF ROLD; |
|  | C24, Col-0. | all others. | TATAAAAT | TATA-BOX | S66544 | PISUM SATIVUM | CORE PROMOTER ELEMENT AROUND -30 OF TRANSCRIPTION START |
|  | C24, Col-0. | all others. | TAAAA | TATA-BOX | L41253 | LYCOPERSICON ESCULENTUM | CORE PROMOTER ELEMENT AROUND -30 OF TRANSCRIPTION START |
| -261 | C24, Col-0. | all others. | TATAAAT | TATABOX2 "TATA BOX"; TATA BOX FOUND IN THE 5'UPSTREAM REGION OF PEA LEGA GENE; SPORAMIN A OF SWEET POTATO; |
|  | C24, Col-0. | all others. | TATAAAT | TATA-BOX | X14597 | PETUNIA HYBRIDA | CORE PROMOTER ELEMENT AROUND -30 OF TRANSCRIPTION START |
|  | C24, Col-0. | all others. | TATAAAT | TATA-BOX | Y13108 | BRASSICA RAPA | CORE PROMOTER ELEMENT AROUND -30 OF TRANSCRIPTION START |
|  | C24, Col-0. | all others. | TATAAAT | TATA-BOX | AC | AC | CORE PROMOTER ELEMENT AROUND -30 OF TRANSCRIPTION START |
|  | C24, Col-0. | all others. | TATAAAT | TATA-BOX | A23331 | ORYZA SATIVA | CORE PROMOTER ELEMENT AROUND -30 OF TRANSCRIPTION START |
|  | C24, Col-0. | all others. | TATAAAT | TATA-BOX | X70333 | BRASSICA OLERACEA | CORE PROMOTER ELEMENT AROUND -30 OF TRANSCRIPTION START |
|  | C24, Col-0. | all others. | TATAAAT | TATA-BOX | U48862 | PETROSELINUM CRISPUM | CORE PROMOTER ELEMENT AROUND -30 OF TRANSCRIPTION START |
|  | C24, Col-0. | all others. | TATAAAT | TATA-BOX | U48863 | PETROSELINUM CRISPUM | CORE PROMOTER ELEMENT AROUND -30 OF TRANSCRIPTION START |
|  | C24, Col-0. | all others. | TATAAAT | TATA-BOX | X67833 | BRASSICA JUNCEA | CORE PROMOTER ELEMENT AROUND -30 OF TRANSCRIPTION START |
| -227 | all others (no data for Ler). | C24, Col-0. | TAATA | TATA-BOX | Z26331 | GLYCINE MAX | CORE PROMOTER ELEMENT AROUND -30 OF TRANSCRIPTION START |
|  | all others (no data for Ler). | C24, Col-0. | ATGAC | SKN-1-LIKE MOTIF | X54314 | ORYZA SATIVA | CIS-ACTING REGULATORY ELEMENT REQUIRED FOR ENDOSPERM EXPRESSION |
| -222 | Ws | C24, Col-0. | CAAAGT | TBOXATGAPB "TBOX" FOUND IN THE ARABIDOPSIS THALIANA (A.T.) GAPB GENE PROMOTER; LOCATED BETWEEN -94 AND -89 (T1) AND ALSO BETWEEN -84 AND -79 (T2); MUTATIONS IN THE "TBOX" RESULTED IN REDUCTIONS OF LIGHT-ACTIVATED GENE TRANSCRIPTION; GAPB ENCODES THE B SUBUNIT OF CHLOROPLAST GLYCERALDEHYDE-3-PHOSPHATE DEHYDROGENASE(GADPH) OF A.T.; |
| -198 | Ws | C24, Col-0. | TAAAG | TAAAGSTKST1 TAAAG MOTIF FOUND IN PROMOTER OF SOLANUM TUBEROSUM (S.T.) KST1 GENE; TARGET SITE FOR TRANS-ACTING STDOF1 PROTEIN CONTROLLING GUARD CELL-SPECIFIC GENE EXPRESSION; KST1 GENE ENCODES A K+ INFLUX CHANNEL OF GUARD CELLS; SEE S000265; |
|  | Ws | C24, Col-0. | TAAAGT | NTBBF1ARROLB NTBBF1(DOF PROTEIN FROM TOBACCO) BINDING SITE IN AGROBACTERIUM RHIZOGENES (A.R.) ROLB GENE; FOUND IN REGULATORY DOMAIN B (-341 TO -306); REQUIRED FOR TISSUE-SPECIFIC EXPRESSION AND AUXIN INDUCTION; |
|  | Ws | C24, Col-0. | AAAG | DOFCOREZM CORE SITE REQUIRED FOR BINDING OF DOF PROTEINS IN MAIZE (Z.M.); DOF PROTEINS ARE DNA BINDING PROTEINS, WITH PRESUMABLY ONLY ONE ZINC FINGER, AND ARE UNIQUE TO PLANTS; FOUR CDNAS ENCODING DOF PROTEINS, DOF1, DOF2, DOF3 AND PBF, HAVE BEEN ISOLATED FROM MAIZE; PBF IS AN ENDOSPERM SPECIFIC DOF PROTEIN THAT BINDS TO PROLAMIN BOX; MAIZE DOF1 ENHANCES TRANSCRIPTION FROM THE PROMOTERS OF BOTH CYTOSOLIC ORTHOPHOSPHATE KINASE (CYPPDK) AND A NON-PHOTOSYNTHETIC PEPC GENE; MAIZE DOF2 SUPRESSED THE C4PEPC PROMOTER; |
|  | Col-0, C24. | Ws | TAAAA | TATA-BOX | L41253 | LYCOPERSICON ESCULENTUM | CORE PROMOTER ELEMENT AROUND -30 OF TRANSCRIPTION START |
| -195 | all others (no data for Ler). | Ws | CTCC | UNNAMED__4 | | PETROSELINUM HORTENSE | |
| -196 | all others (no data for Ler). | Ws | CTCC | UNNAMED__4 | | PETROSELINUM HORTENSE | |
| -177 | No | all others (no data for Ler). | CTCATTTT | INRNTPSADB "INR (INITIATER)" ELEMENTS FOUND IN THE TOBACCO PSADB GENE PROMOTER WITHOUT TATA BOXES; LIGHT-RESPONSIVE TRANSCRIPTION OF PSADB DEPENDS ON INR, BUT NOT TATA BOX; |
|  | all others (no data for Ler). | No | CTCATTTC | INRNTPSADB "INR (INITIATER)" ELEMENTS FOUND IN THE TOBACCO PSADB GENE PROMOTER WITHOUT TATA BOXES; LIGHT-RESPONSIVE TRANSCRIPTION OF PSADB DEPENDS ON INR, BUT NOT TATA BOX; |
|  | No | all others (no data for Ler). | TTTTA | TATA-BOX | L41253 | LYCOPERSICON ESCULENTUM | CORE PROMOTER ELEMENT AROUND -30 OF TRANSCRIPTION START |
| -166 | No | all others (no data for Ler). | TTTAAAAA | TATA-BOX | U45858 | ZEA MAYS | CORE PROMOTER ELEMENT AROUND -30 OF TRANSCRIPTION START |
|  | all others (no data for Ler). | No | TATTCT | -10PEHVPSBD "-10 PROMOTER ELEMENT" FOUND IN THE BARLEY (H.V.) CHLOROPLAST PSBD GENE PROMOTER; INVOLVED IN THE EXPRESSION OF THE PLASTID GENE PSBD WHICH ENCODES A PHOTOSYSTEM II REACTION CENTER CHLOROPHYLL-BINDING PROTEIN THAT IS ACTIVATED BY BLUE, WHITE O |
|  | No | all others (no data for Ler). | TTTTA | TATA-BOX | L41253 | LYCOPERSICON ESCULENTUM | CORE PROMOTER ELEMENT AROUND -30 OF TRANSCRIPTION START |
| -159 | all others (no data for Ler). | Ws | AAAG | DOFCOREZM CORE SITE REQUIRED FOR BINDING OF DOF PROTEINS IN MAIZE (Z.M.); DOF PROTEINS ARE DNA BINDING PROTEINS, WITH PRESUMABLY ONLY ONE ZINC FINGER, AND ARE UNIQUE TO PLANTS; FOUR CDNAS ENCODING DOF PROTEINS, DOF1, DOF2, DOF3 AND PBF, HAVE BEEN ISOLATED FROM MAIZE; PBF IS AN ENDOSPERM SPECIFIC DOF PROTEIN THAT BINDS TO PROLAMIN BOX; MAIZE DOF1 ENHANCES TRANSCRIPTION FROM THE PROMOTERS OF BOTH CYTOSOLIC ORTHOPHOSPHATE KINASE (CYPPDK) AND A NON-PHOTOSYNTHETIC PEPC GENE; MAIZE DOF2 SUPRESSED THE C4PEPC PROMOTER; |
| -99 | all others (no data for Ler). | Col-0, C24. | CACCTG | RAV1BAT BINDING CONSENSUS SEQUENCE OF AN ARABIDOPSIS (A.T.) TRANSCRIPTION FACTOR, RAV1; RAV1 SPECIFICALLY BINDS TO DNA WITH BIPARTITE SEQUENCE MOTIFS OF RAV1-A (CAACA) AND RAV1-B (CACCTG); RAV1 PROTEIN CONTAIN AP2-LIKE AND B3-LIKE DOMAINS; THE AP2-LIKE AND B3-LIKE DOMAINS RECOGNIZE THE CAACA AND CACCTG MOTIFS, RESPECTIVELY; THE EXPRESSION LEVEL OF RAV1 WERE RELATIVELY HIGH IN ROSETTE LEAVES AND ROOTS; SEE S000314(CAACA); |
|  | all others (no data for Ler). | Col-0, C24. | CAAT | CAAT-BOX | X78205 | HORDEUM VULGARE | COMMON CIS-ACTING ELEMENT IN PROMOTER AND ENHANCER REGIONS |
|  | all others (no data for Ler). | Col-0, C24. | AAACCA | ARE | U45858 | ZEA MAYS | CIS-ACTING REGULATORY ELEMENT ESSENTIAL FOR THE ANAEROBIC INDUCTION |
|  | all others (no data for Ler). | Col-0, C24. | AAACCA | ARE | U45859 | ZEA MAYS | CIS-ACTING REGULATORY ELEMENT ESSENTIAL FOR THE ANAEROBIC INDUCTION |
|  | all others (no data for Ler). | Col-0, C24. | CAAT | CAAT-BOX | S44160 | LYCOPERSICON ESCULENTUM | COMMON CIS-ACTING ELEMENT IN PROMOTER AND ENHANCER REGIONS |
|  | all others (no data for Ler). | Col-0, C24. | AGAAA | POLLEN1LELAT52 ONE OF TWO CO-DEPENDENT REGULATORY ELEMENTS RESPONSIBLE FOR POLLEN SPECIFIC ACTIVATION OF TOMATO (L.E.) LAT52 GENE; FOUND AT -72 TO -68 REGION; SEE S000246 (POLLEN2LELAT52); AGAAA AND TCCACCATA (S000246) ARE REQUIRED FOR POLLEN SPECIFIC EXPRESSION; |
|  | all others (no data for Ler). | Col-0, C24. | CAAT | CAATBOX1 "CAAT PROMOTER CONSENSUS SEQUENCE" FOUND IN LEGA GENE OF PEA; |
|  | all others (no data for Ler). | Col-0, C24. | CAAT | CAAT-BOX | L02124 | NICOTIANA TABACUM | COMMON CIS-ACTING ELEMENT IN PROMOTER AND ENHANCER REGIONS |
|  | all others (no data for Ler). | Col-0, C24. | CCACCTGG | SITEIOSPCNA "SITE I" OF RICE (O.S.) PCNA (PROLIFERATING CELL NUCLEAR ANTIGEN) GENE; FOUND AT -201 TO -194; RESEMBLE G-BOX; MAY CONTRIBUTE IN PART TO TRANSCRIPTIONAL ACTIVATION; |
|  | all others (no data for Ler). | Col-0, C24. | CACCTG | EBOXBNNAPA E-BOX OF NAPA STORAGE-PROTEIN GENE OF BRASSICA NAPUS (B.N.); SEE S000042 (CACGTGMOTIF); |
| -86 | all others (no data for Ler). | WS | CCAAT | CAAT-BOX | L41253 | LYCOPERSICON ESCULENTUM | COMMON CIS-ACTING ELEMENT IN PROMOTER AND ENHANCER REGIONS |
|  | all others (no data for Ler). | WS | CCAAT | CAAT-BOX | Y13535 | BRASSICA OLERACEA | COMMON CIS-ACTING ELEMENT IN PROMOTER AND ENHANCER REGIONS |
|  | all others (no data for Ler). | WS | CCAAT | CAAT-BOX | D45890 | ORYZA SATIVA | COMMON CIS-ACTING ELEMENT IN PROMOTER AND ENHANCER REGIONS |
|  | all others (no data for Ler). | WS | CCAAT | CAAT-BOX | X98521 | BRASSICA OLERACEA | COMMON CIS-ACTING ELEMENT IN PROMOTER AND ENHANCER REGIONS |
|  | all others (no data for Ler). | WS | CCAAT | CAAT-BOX | Z13987 | SOLANUM TUBEROSUM | COMMON CIS-ACTING ELEMENT IN PROMOTER AND ENHANCER REGIONS |
|  | all others (no data for Ler). | WS | CCAAT | CAAT-BOX | U45858 | ZEA MAYS | COMMON CIS-ACTING ELEMENT IN PROMOTER AND ENHANCER REGIONS |
|  | all others (no data for Ler). | WS | CCAAT | CAAT-BOX | Z35160 | SOLANUM TUBEROSUM | COMMON CIS-ACTING ELEMENT IN PROMOTER AND ENHANCER REGIONS |
|  | all others (no data for Ler). | WS | AATGGAAATG | MRNA3ENDTAH3 CIS ELEMENT IN 3' END REGION OF WHEAT (T.A.) HISTONE H3 MRNA; 3' END FORMATION; ALSO FOUND IN HISTONE GENES OF OTHER PLANTS, YEAST, ETC; |
|  | all others (no data for Ler). | WS | CCAAT | CAAT-BOX | D13044 | ARABIDOPSIS THALIANA | COMMON CIS-ACTING ELEMENT IN PROMOTER AND ENHANCER REGIONS |
|  | all others (no data for Ler). | WS | CCAAT | CCAATBOX1 COMMON SEQUENCE FOUND IN THE 5'-NON-CODING REGIONS OF EUKARYOTIC GENES; |
| -85 | C24, Col-0. | all others (no data for Ler). | CGTGG | UNNAMED__1 | | ZEA MAYS | |
|  | C24, Col-0. | all others (no data for Ler). | CGTGG | UNNAMED__3 | | ZEA MAYS | |
| -40 | all others (no data for Ler). | Ws | TTTTTC | GT1CONSENSUS CONSENSUS GT-1 BINDING SITE IN MANY LIGHT-REGULATED GENES, E.G., RBCS FROM MANY SPECIES, PHYA FROM OAT AND RICE, SPINACH RCA AND PETA, AND BEAN CHS15; R=A/G; W=A/T; FOR A COMPILATION OF RELATED GT ELEMENTS AND FACTORS, SEE VILLAIN ET AL. (1996); GT-1 CAN STABILIZE THE TFIIA-TBP-DNA (TATA BOX) COMPLEX; THE ACTIVATION MECHANISM OF GT-1 MAY BE ACHIEVED THROUGH DIRECT INTERACTION BETWEEN TFIIA AND GT-1; BINDING OF GT-1-LIKE FACTORS TO THE PR-1A PROMOTER INFLUENCES THE LEVEL OF SA-INDUCIBLE GENE EXPRESSION; |
|  | all others (no data for Ler). | Ws | TTTTTTCC | PYRIMIDINEBOXHVEPB1 "PYRIMIDINE BOX" FOUND IN THE BARLEY (H.V.) EPB-1 (CYSTEINE PROTEINASE) GENE PROMOTER; LOCATED BETWEEN -120 TO -113; REQUIRED FOR GA INDUCTION; SEE S000297, S000259; |
|  | all others (no data for Ler). | Ws | CTCC | UNNAMED__4 | | PETROSELINUM HORTENSE | |
|  |  |  |  |  |
| At2g32930 CCCH Zn-finger protein | | | | |
|  |  |  |  |  |
| -236 | Ws | others | CAAT | CAAT-BOX | X78205 | HORDEUM VULGARE | COMMON CIS-ACTING ELEMENT IN PROMOTER AND ENHANCER REGIONS |
|  | Ws | others | CAAT | CAAT-BOX | S44160 | LYCOPERSICON ESCULENTUM | COMMON CIS-ACTING ELEMENT IN PROMOTER AND ENHANCER REGIONS |
|  | Ws | others | TTCAATTT | INRNTPSADB "INR (INITIATER)" ELEMENTS FOUND IN THE TOBACCO PSADB GENE PROMOTER WITHOUT TATA BOXES; LIGHT-RESPONSIVE TRANSCRIPTION OF PSADB DEPENDS ON INR, BUT NOT TATA BOX; |
|  | Ws | others | CAAT | CAATBOX1 "CAAT PROMOTER CONSENSUS SEQUENCE" FOUND IN LEGA GENE OF PEA; |
|  | Ws | others | CAAT | CAAT-BOX | L02124 | NICOTIANA TABACUM | COMMON CIS-ACTING ELEMENT IN PROMOTER AND ENHANCER REGIONS |
|  | Ws | others | CAATT | CAAT-BOX | Z26331 | GLYCINE MAX | COMMON CIS-ACTING ELEMENT IN PROMOTER AND ENHANCER REGIONS |
|  |  |  |  |  |
| At2g34290 putative protein kinase | | | | |
|  |  |  |  |  |
| -90 | all others | No-0. | TGGTTT | ARE | U45858 | ZEA MAYS | CIS-ACTING REGULATORY ELEMENT ESSENTIAL FOR THE ANAEROBIC INDUCTION |
|  | all others | No-0. | TTGGTT | REALPHALGLHCB21 "REALPHA" FOUND IN LEMNA GIBBA LHCB21 GENE PROMOTER; LOCATED AT -134 TO -129; BINDING SITE OF PROTEINS OF WHOLE-CELL EXTRACTS; THE DNA BINDNIG ACTIVITY IS HIGH IN ETIOLATED PLANTS BUT MUCH LOWER IN GREEN PLANTS; REQUIRED FOR PHYTOCHROME REGULATION; SEE S000363; |
|  | all others | No-0. | TGGTTT | ARE | U45859 | ZEA MAYS | CIS-ACTING REGULATORY ELEMENT ESSENTIAL FOR THE ANAEROBIC INDUCTION |
|  | No-0. | all others | ATTTCC | GT1CONSENSUS CONSENSUS GT-1 BINDING SITE IN MANY LIGHT-REGULATED GENES, E.G., RBCS FROM MANY SPECIES, PHYA FROM OAT AND RICE, SPINACH RCA AND PETA, AND BEAN CHS15; R=A/G; W=A/T; FOR A COMPILATION OF RELATED GT ELEMENTS AND FACTORS, SEE VILLAIN ET AL. (1996); GT-1 CAN STABILIZE THE TFIIA-TBP-DNA (TATA BOX) COMPLEX; THE ACTIVATION MECHANISM OF GT-1 MAY BE ACHIEVED THROUGH DIRECT INTERACTION BETWEEN TFIIA AND GT-1; BINDING OF GT-1-LIKE FACTORS TO THE PR-1A PROMOTER INFLUENCES THE LEVEL OF SA-INDUCIBLE GENE EXPRESSION; |
|  |  |  |  |  |
| At3g13445 transcription initiation factor TFIID-1 (TATA sequence-binding protein 1) | | | | |
|  |  |  |  |  |
| -1210 | No-0. | others | TTGGTT | REALPHALGLHCB21 "REALPHA" FOUND IN LEMNA GIBBA LHCB21 GENE PROMOTER; LOCATED AT -134 TO -129; BINDING SITE OF PROTEINS OF WHOLE-CELL EXTRACTS; THE DNA BINDNIG ACTIVITY IS HIGH IN ETIOLATED PLANTS BUT MUCH LOWER IN GREEN PLANTS; REQUIRED FOR PHYTOCHROME REGULATION; SEE S000363; |
|  | No-0. | others | GGTTGG | MYBPZM CORE OF CONSENSUS MAIZE P (MYB HOMOLOG) BINDING SITE; W=A/T; 6 BP CORE; MAIZE P GENE SPECIFIES RED PIGMENTATION OF KERNEL PERICARP, COB, AND OTHER FLORAL ORGANS; P BINDS TO A1 GENE, BUT NOT BZ1 GENE; MAIZE C1 (MYB HOMOLOG) ACTIVATES BOTH A1 AND BZ1 GENES (GROTEWOLD ET AL. 1994); W=A/T; |
|  | No-0. | others | GGTTGGTT | MYBPLANT PLANT MYB BINDING SITE; CONSENSUS SEQUENCE RELATED TO BOX P IN PROMOTERS OF PHENYLPROPANOID BIOSYNTHETIC GENES SUCH AS PAL, CHS, CHI, DFR, CL, BZ1; MYB305; M=A/C; W=A/T; SEE S000355; THE AMMYB308 AND AMMYB330 TRANSCRIPTION FACTORS FROM ANTIRRHINUM MAJUS REGULATE PHENYLPROPANOID AND LIGNIN BIOSYNTHESIS IN TRANSGENIC TOBACCO; |
| -865 | Ws, No-0 | others | GTCAT | SKN-1-LIKE MOTIF | X54314 | ORYZA SATIVA | CIS-ACTING REGULATORY ELEMENT REQUIRED FOR ENDOSPERM EXPRESSION |
| -697 | Ws | others | AACGTT | ACGTTBOX "T-BOX" ACCORDING TO THE NOMENCLATURE OF ACGT ELEMENTS BY FOSTER ET AL. (FASEB J 8:192-200 (1994)); ONE OF ACGT ELEMENTS; SEE ALSO ACGTABOX (S000130), ACGTCBOX (S000131), AND CACGTGMOTIF (S000042); |
| -655 | others | Ws, No-0 | CTTT | DOFCOREZM CORE SITE REQUIRED FOR BINDING OF DOF PROTEINS IN MAIZE (Z.M.); DOF PROTEINS ARE DNA BINDING PROTEINS, WITH PRESUMABLY ONLY ONE ZINC FINGER, AND ARE UNIQUE TO PLANTS; FOUR CDNAS ENCODING DOF PROTEINS, DOF1, DOF2, DOF3 AND PBF, HAVE BEEN ISOLATED FROM MAIZE; PBF IS AN ENDOSPERM SPECIFIC DOF PROTEIN THAT BINDS TO PROLAMIN BOX; MAIZE DOF1 ENHANCES TRANSCRIPTION FROM THE PROMOTERS OF BOTH CYTOSOLIC ORTHOPHOSPHATE KINASE (CYPPDK) AND A NON-PHOTOSYNTHETIC PEPC GENE; MAIZE DOF2 SUPRESSED THE C4PEPC PROMOTER; |
| -626 | others | Ws, No-0 | GTCAT | SKN-1-LIKE MOTIF | X54314 | ORYZA SATIVA | CIS-ACTING REGULATORY ELEMENT REQUIRED FOR ENDOSPERM EXPRESSION |
|  | Ws, No-0 | others | TCAC | GTGANTG10 "GTGA MOTIF" FOUND IN THE PROMOTER OF THE TABACCO (N.T.) LATE POLLEN GENE G10 WHICH SHOWS HOMOLOGY TO PECTATE LYASE AND IS THE PUTATIVE HOMOLOGUE OF THE TOMATO GENE LAT56; LOCATED BETWEEN -96 AND -93; SEE S000280; |
|  |  |  |  |  |
| At4g10160 Putative RING Zn-finger protein | | | | |
|  |  |  |  |  |
| -1103 | col-0 | others | TAAAAAT | SEF4MOTIFGM7S "SEF4 BINDING SITE"; SOYBEAN (G.M.) CONSENSUS SEQUENCE FOUND IN 5'UPSTREAM REGION (-199) OF BETA-CONGLYCININ (7S GLOBULIN) GENE (GMG17.1); "BINDING WITH SEF4 (SOYBEAN EMBRYO FACTOR 4)"; R=A/G; |
|  | others | col-0 | TATTTAAA | TATA-BOX | X84728 | ARABIDOPSIS THALIANA | CORE PROMOTER ELEMENT AROUND -30 OF TRANSCRIPTION START |
|  | Col-0 | others | TTTTA | TATA-BOX | L41253 | LYCOPERSICON ESCULENTUM | CORE PROMOTER ELEMENT AROUND -30 OF TRANSCRIPTION START |
|  | others | Col-0 | TATTTAA | TATABOXOSPAL BINDING SITE FOR OSTBP2, FOUND IN THE PROMOTER OF RICE PAL GENE ENCODING PHENYLALANINE AMMONIA-LYASE; OSTFIIB STIMULATED THE DNA BINDING AND BENDING ACTIVITIES OF OSTBP2 AND SYNERGISTICALLY ENHANCED OSTBP2-MEDIATED TRANSCRIPTION FROM THE PAL PROMOTER; |
| -985 | others | Ws | ATTG | CAAT-BOX | X78205 | HORDEUM VULGARE | COMMON CIS-ACTING ELEMENT IN PROMOTER AND ENHANCER REGIONS |
|  | others | Ws | TATAAT | TATA-BOX | U27107 | BRASSICA NAPUS | CORE PROMOTER ELEMENT AROUND -30 OF TRANSCRIPTION START |
|  | others | Ws | ATTG | CAAT-BOX | S44160 | LYCOPERSICON ESCULENTUM | COMMON CIS-ACTING ELEMENT IN PROMOTER AND ENHANCER REGIONS |
|  | others | Ws | ATTG | CAATBOX1 "CAAT PROMOTER CONSENSUS SEQUENCE" FOUND IN LEGA GENE OF PEA; |
|  | others | Ws | ATTG | CAAT-BOX | L02124 | NICOTIANA TABACUM | COMMON CIS-ACTING ELEMENT IN PROMOTER AND ENHANCER REGIONS |
|  | others | Ws | AATTG | CAAT-BOX | Z26331 | GLYCINE MAX | COMMON CIS-ACTING ELEMENT IN PROMOTER AND ENHANCER REGIONS |
|  | others | Ws | TATAAT | TATA-BOX | U46217 | PETROSELINUM CRISPUM | CORE PROMOTER ELEMENT AROUND -30 OF TRANSCRIPTION START |
| -830 | No-0. | others | TGGTTT | ARE | U45858 | ZEA MAYS | CIS-ACTING REGULATORY ELEMENT ESSENTIAL FOR THE ANAEROBIC INDUCTION |
|  | No-0. | others | TTGGTT | REALPHALGLHCB21 "REALPHA" FOUND IN LEMNA GIBBA LHCB21 GENE PROMOTER; LOCATED AT -134 TO -129; BINDING SITE OF PROTEINS OF WHOLE-CELL EXTRACTS; THE DNA BINDNIG ACTIVITY IS HIGH IN ETIOLATED PLANTS BUT MUCH LOWER IN GREEN PLANTS; REQUIRED FOR PHYTOCHROME REGULATION; SEE S000363; |
|  | No-0. | others | TGGTTT | ARE | U45859 | ZEA MAYS | CIS-ACTING REGULATORY ELEMENT ESSENTIAL FOR THE ANAEROBIC INDUCTION |
|  | No-0. | others | GTTTGGTT | MYBPLANT PLANT MYB BINDING SITE; CONSENSUS SEQUENCE RELATED TO BOX P IN PROMOTERS OF PHENYLPROPANOID BIOSYNTHETIC GENES SUCH AS PAL, CHS, CHI, DFR, CL, BZ1; MYB305; M=A/C; W=A/T; SEE S000355; THE AMMYB308 AND AMMYB330 TRANSCRIPTION FACTORS FROM ANTIRRHINUM MAJUS REGULATE PHENYLPROPANOID AND LIGNIN BIOSYNTHESIS IN TRANSGENIC TOBACCO; |
| -485 | Ws | others | CTCATTCC | INRNTPSADB "INR (INITIATER)" ELEMENTS FOUND IN THE TOBACCO PSADB GENE PROMOTER WITHOUT TATA BOXES; LIGHT-RESPONSIVE TRANSCRIPTION OF PSADB DEPENDS ON INR, BUT NOT TATA BOX; |
| -400 | others | Ws | TTTAATT | POLASIG2 "POLYA SIGNAL"; POLY A SIGNAL FOUND IN RICE ALPHA-AMYLASE; -10 TO -30 IN THE CASE OF ANIMAL GENES. AATAAA; AATAAT; AATTAAA; AATAAG; |
| -226 | No-0, La-er, Ws | Col-0, C24. | TATC | GATABOX "GATA BOX"; GATA MOTIF IN CAMV 35S PROMOTER; BINDING WITH ASF-2; THREE GATA BOX REPEATS WERE FOUND IN THE PROMOTER OF PETUNIA (P.H.) CHLOROPHYLL A/B BINDING PROTEIN, CAB22 GENE; REQUIRED FOR HIGH LEVEL, LIGHT REGULATED, AND TISSUE SPECIFIC EXPRESSION; CONSERVED IN THE PROMOTER OF ALL LHCII TYPE I CAB GENES; |
|  | Col-0, C24. | No-0, La-er, Ws | ATATT | ROOTMOTIFTAPOX1 MOTIF FOUND BOTH IN PROMOTERS OF ROLD; |
| -164 | others | La-er | ATATAA | TATA-BOX | Y13535 | BRASSICA OLERACEA | CORE PROMOTER ELEMENT AROUND -30 OF TRANSCRIPTION START |
|  | others | La-er | TATA | TATA-BOX | Y10182 | CATHARANTHUS ROSEUS | CORE PROMOTER ELEMENT AROUND -30 OF TRANSCRIPTION START |
|  | La-er | others | CAACA | RAV1AAT BINDING CONSENSUS SEQUENCE OF ARABIDOPSIS (A.T.) TRANSCRIPTION FACTOR, RAV1; RAV1 SPECIFICALLY BINDS TO DNA WITH BIPARTITE SEQUENCE MOTIFS OF RAV1-A (CAACA) AND RAV1-B (CACCTG); RAV1 PROTEIN CONTAIN AP2-LIKE AND B3-LIKE DOMAINS; THE AP2-LIKE AND B3-LIKE DOMAINS RECOGNIZE THE CAACA AND CACCTG MOTIFS, RESPECTIVELY; THE EXPRESSION LEVEL OF RAV1 WERE RELATIVELY HIGH IN ROSETTE LEAVES AND ROOTS; SEE S000315(CACCTG); |
|  | others | La-er | TATA | TATA-BOX | AJ003135 | ARABIDOPSIS THALIANA | CORE PROMOTER ELEMENT AROUND -30 OF TRANSCRIPTION START |
|  | others | La-er | TATA | TATA-BOX | M92353 | ARABIDOPSIS THALIANA | CORE PROMOTER ELEMENT AROUND -30 OF TRANSCRIPTION START |
|  | others | La-er | TATAA | TATA-BOX | D13044 | ARABIDOPSIS THALIANA | CORE PROMOTER ELEMENT AROUND -30 OF TRANSCRIPTION START |
|  |  |  |  |  |
| At4g39410 WRKY family transcription factor | | | | |
|  |  |  |  |  |
| -858 | others | No-0 | CAACA | RAV1AAT BINDING CONSENSUS SEQUENCE OF ARABIDOPSIS (A.T.) TRANSCRIPTION FACTOR, RAV1; RAV1 SPECIFICALLY BINDS TO DNA WITH BIPARTITE SEQUENCE MOTIFS OF RAV1-A (CAACA) AND RAV1-B (CACCTG); RAV1 PROTEIN CONTAIN AP2-LIKE AND B3-LIKE DOMAINS; THE AP2-LIKE AND B3-LIKE DOMAINS RECOGNIZE THE CAACA AND CACCTG MOTIFS, RESPECTIVELY; THE EXPRESSION LEVEL OF RAV1 WERE RELATIVELY HIGH IN ROSETTE LEAVES AND ROOTS; SEE S000315(CACCTG); |
|  | No-0 | others | TCAC | GTGANTG10 "GTGA MOTIF" FOUND IN THE PROMOTER OF THE TABACCO (N.T.) LATE POLLEN GENE G10 WHICH SHOWS HOMOLOGY TO PECTATE LYASE AND IS THE PUTATIVE HOMOLOGUE OF THE TOMATO GENE LAT56; LOCATED BETWEEN -96 AND -93; SEE S000280; |
| -454 | No-0 | others | ATTTTTA | SEF4MOTIFGM7S "SEF4 BINDING SITE"; SOYBEAN (G.M.) CONSENSUS SEQUENCE FOUND IN 5'UPSTREAM REGION (-199) OF BETA-CONGLYCININ (7S GLOBULIN) GENE (GMG17.1); "BINDING WITH SEF4 (SOYBEAN EMBRYO FACTOR 4)"; R=A/G; |
|  | others | No-0 | TAAAAAT | SEF4MOTIFGM7S "SEF4 BINDING SITE"; SOYBEAN (G.M.) CONSENSUS SEQUENCE FOUND IN 5'UPSTREAM REGION (-199) OF BETA-CONGLYCININ (7S GLOBULIN) GENE (GMG17.1); "BINDING WITH SEF4 (SOYBEAN EMBRYO FACTOR 4)"; R=A/G; |
| **At2g18790 Phytochrome B (PHYB)** | | | | |
| -965 | WS | others | TTCTAT | BOXIINTPATPB "BOX II" FOUND IN THE TOBACCO (N.T.) PLASTID ATPB GENE PROMOTER; CONSERVED IN SEVERAL NCII (NONCONSENSUS TYPE II) PROMOTERS OF PLASTID GENES; IMPORTANT FOR THE ACTIVITY OF THIS NCII PROMOTER; SEE S000295; |
|  | WS | others | ATTTTC | GT1CONSENSUS CONSENSUS GT-1 BINDING SITE IN MANY LIGHT-REGULATED GENES, E.G., RBCS FROM MANY SPECIES, PHYA FROM OAT AND RICE, SPINACH RCA AND PETA, AND BEAN CHS15; R=A/G; W=A/T; FOR A COMPILATION OF RELATED GT ELEMENTS AND FACTORS, SEE VILLAIN ET AL. (1996); GT-1 CAN STABILIZE THE TFIIA-TBP-DNA (TATA BOX) COMPLEX; THE ACTIVATION MECHANISM OF GT-1 MAY BE ACHIEVED THROUGH DIRECT INTERACTION BETWEEN TFIIA AND GT-1; BINDING OF GT-1-LIKE FACTORS TO THE PR-1A PROMOTER INFLUENCES THE LEVEL OF SA-INDUCIBLE GENE EXPRESSION; |
|  | others | WS | TTTTTC | GT1CONSENSUS CONSENSUS GT-1 BINDING SITE IN MANY LIGHT-REGULATED GENES, E.G., RBCS FROM MANY SPECIES, PHYA FROM OAT AND RICE, SPINACH RCA AND PETA, AND BEAN CHS15; R=A/G; W=A/T; FOR A COMPILATION OF RELATED GT ELEMENTS AND FACTORS, SEE VILLAIN ET AL. (1996); GT-1 CAN STABILIZE THE TFIIA-TBP-DNA (TATA BOX) COMPLEX; THE ACTIVATION MECHANISM OF GT-1 MAY BE ACHIEVED THROUGH DIRECT INTERACTION BETWEEN TFIIA AND GT-1; BINDING OF GT-1-LIKE FACTORS TO THE PR-1A PROMOTER INFLUENCES THE LEVEL OF SA-INDUCIBLE GENE EXPRESSION; |
| -938 | WS | others | TATC | GATABOX "GATA BOX"; GATA MOTIF IN CAMV 35S PROMOTER; BINDING WITH ASF-2; THREE GATA BOX REPEATS WERE FOUND IN THE PROMOTER OF PETUNIA (P.H.) CHLOROPHYLL A/B BINDING PROTEIN, CAB22 GENE; REQUIRED FOR HIGH LEVEL, LIGHT REGULATED, AND TISSUE SPECIFIC EXPRESSION; CONSERVED IN THE PROMOTER OF ALL LHCII TYPE I CAB GENES; |
|  | others | WS | CTTT | DOFCOREZM CORE SITE REQUIRED FOR BINDING OF DOF PROTEINS IN MAIZE (Z.M.); DOF PROTEINS ARE DNA BINDING PROTEINS, WITH PRESUMABLY ONLY ONE ZINC FINGER, AND ARE UNIQUE TO PLANTS; FOUR CDNAS ENCODING DOF PROTEINS, DOF1, DOF2, DOF3 AND PBF, HAVE BEEN ISOLATED FROM MAIZE; PBF IS AN ENDOSPERM SPECIFIC DOF PROTEIN THAT BINDS TO PROLAMIN BOX; MAIZE DOF1 ENHANCES TRANSCRIPTION FROM THE PROMOTERS OF BOTH CYTOSOLIC ORTHOPHOSPHATE KINASE (CYPPDK) AND A NON-PHOTOSYNTHETIC PEPC GENE; MAIZE DOF2 SUPRESSED THE C4PEPC PROMOTER; |
| -754 | No-0 and WS | others | TATA | TATA-BOX | Y10182 | CATHARANTHUS ROSEUS | CORE PROMOTER ELEMENT AROUND -30 OF TRANSCRIPTION START |
|  | No-0 and WS | others | TATA | TATA-BOX | AJ003135 | ARABIDOPSIS THALIANA | CORE PROMOTER ELEMENT AROUND -30 OF TRANSCRIPTION START |
|  | No-0 and WS | others | TATA | TATA-BOX | M92353 | ARABIDOPSIS THALIANA | CORE PROMOTER ELEMENT AROUND -30 OF TRANSCRIPTION START |
|  | No-0 and WS | others | AATAT | ROOTMOTIFTAPOX1 MOTIF FOUND BOTH IN PROMOTERS OF ROLD; |
| -445 | Col-0, C24, Ler | WS, No-0. | AAACCA | ARE | U45858 | ZEA MAYS | CIS-ACTING REGULATORY ELEMENT ESSENTIAL FOR THE ANAEROBIC INDUCTION |
|  | Col-0, C24, Ler | WS, No-0. | AAACCA | ARE | U45859 | ZEA MAYS | CIS-ACTING REGULATORY ELEMENT ESSENTIAL FOR THE ANAEROBIC INDUCTION |
|  | No-0 and WS | Col-0, C24, Ler | TAAAG | TAAAGSTKST1 TAAAG MOTIF FOUND IN PROMOTER OF SOLANUM TUBEROSUM (S.T.) KST1 GENE; TARGET SITE FOR TRANS-ACTING STDOF1 PROTEIN CONTROLLING GUARD CELL-SPECIFIC GENE EXPRESSION; KST1 GENE ENCODES A K+ INFLUX CHANNEL OF GUARD CELLS; SEE S000265; |
|  | No-0 and WS | Col-0, C24, Ler | AAAG | DOFCOREZM CORE SITE REQUIRED FOR BINDING OF DOF PROTEINS IN MAIZE (Z.M.); DOF PROTEINS ARE DNA BINDING PROTEINS, WITH PRESUMABLY ONLY ONE ZINC FINGER, AND ARE UNIQUE TO PLANTS; FOUR CDNAS ENCODING DOF PROTEINS, DOF1, DOF2, DOF3 AND PBF, HAVE BEEN ISOLATED FROM MAIZE; PBF IS AN ENDOSPERM SPECIFIC DOF PROTEIN THAT BINDS TO PROLAMIN BOX; MAIZE DOF1 ENHANCES TRANSCRIPTION FROM THE PROMOTERS OF BOTH CYTOSOLIC ORTHOPHOSPHATE KINASE (CYPPDK) AND A NON-PHOTOSYNTHETIC PEPC GENE; MAIZE DOF2 SUPRESSED THE C4PEPC PROMOTER; |
| -62 | WS | others | TTCACTCT | INRNTPSADB "INR (INITIATER)" ELEMENTS FOUND IN THE TOBACCO PSADB GENE PROMOTER WITHOUT TATA BOXES; LIGHT-RESPONSIVE TRANSCRIPTION OF PSADB DEPENDS ON INR, BUT NOT TATA BOX; |
| -618 | WS | others | ATTG | CAAT-BOX | X78205 | HORDEUM VULGARE | COMMON CIS-ACTING ELEMENT IN PROMOTER AND ENHANCER REGIONS |
|  | WS | others | ATTG | CAAT-BOX | S44160 | LYCOPERSICON ESCULENTUM | COMMON CIS-ACTING ELEMENT IN PROMOTER AND ENHANCER REGIONS |
|  | others | WS | ATTTG | CAAT-BOX | Y13108 | BRASSICA RAPA | COMMON CIS-ACTING ELEMENT IN PROMOTER AND ENHANCER REGIONS |
|  | others | WS | ATTTGTAT | ATGCAAAT-MOTIF | X52153 | ORYZA SATIVA | CIS-ACTING REGULATORY ELEMENT ASSOCIATED TO THE TGAGTCA MOTIF |
|  | WS | others | ATTG | CAATBOX1 "CAAT PROMOTER CONSENSUS SEQUENCE" FOUND IN LEGA GENE OF PEA; |
|  | WS | others | ATTG | CAAT-BOX | L02124 | NICOTIANA TABACUM | COMMON CIS-ACTING ELEMENT IN PROMOTER AND ENHANCER REGIONS |
|  | others | WS | ATTTG | CAAT-BOX | D10661 | PISUM SATIVUM | COMMON CIS-ACTING ELEMENT IN PROMOTER AND ENHANCER REGIONS |
