# Supplementary figures and images for: Contribution of transcriptional regulation to natural variations in Arabidopsis
Source: Genome Biol. 2005 Mar 15;6(4):R32. doi: 10.1186/gb-2005-6-4-r32 (PMC1088960; doi:10.1186/gb-2005-6-4-r32)

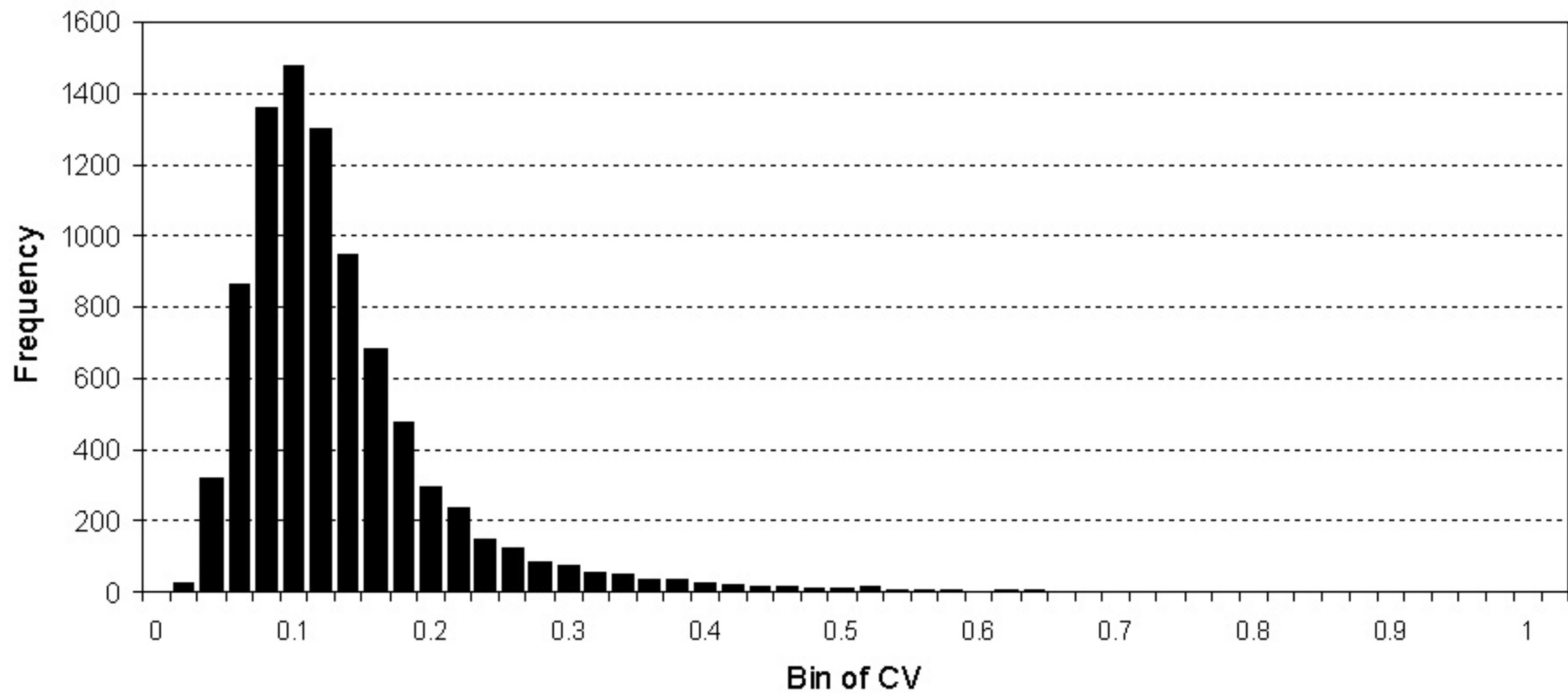

Supplement: Additional File 8 — A figure showing a histogram of coefficient of variance (CV) based on genomic hybridization intensity indices from the five accessions [file gb-2005-6-4-r32-S8.pdf]
